# Nubian Levallois technology associated with southernmost Neanderthals

**DOI:** 10.1038/s41598-021-82257-6

**Published:** 2021-02-15

**Authors:** James Blinkhorn, Clément Zanolli, Tim Compton, Huw S. Groucutt, Eleanor M. L. Scerri, Lucile Crété, Chris Stringer, Michael D. Petraglia, Simon Blockley

**Affiliations:** 1 Pan-African Evolution Research Group, Max Planck Institute for the Science of Human History, Kahlaische Strasse 10, 07745 Jena, Germany; 2 Centre for Quaternary Research, Department of Geography, Royal Holloway University of London, Egham Hill, Egham, Surrey UK; 3 Univ. Bordeaux, CNRS, MCC, PACEA, UMR 5199, 33600 Pessac, France; 4 Centre for Human Evolution Research, Department of Earth Sciences, Natural History Museum, Cromwell Road, London, SW7 5BD UK; 5 Extreme Events Research Group, Max Planck Institutes for Chemical Ecology, The Science of Human History, and Biogeochemistry, Hans-Knöll-Strasse 8, 07745 Jena, Germany; 6 Department of Archaeology, Max Planck Institute for the Science of Human History, Kahlaische Strasse 10, 07745 Jena, Germany; 7 Department of Classics and Archaeology, University of Malta, Msida, 2080 MSD Malta; 8 Human Origins Program, Smithsonian Institution, Washington, DC 20560 USA; 9 School of Social Science, The University of Queensland, Brisbane, QLD 4072 Australia; 10 Institute of Prehistoric Archaeology, University of Cologne, 50931 Cologne, Germany

**Keywords:** Archaeology, Biological anthropology

## Abstract

Neanderthals occurred widely across north Eurasian landscapes, but between ~ 70 and 50 thousand years ago (ka) they expanded southwards into the Levant, which had previously been inhabited by *Homo sapiens*. Palaeoanthropological research in the first half of the twentieth century demonstrated alternate occupations of the Levant by Neanderthal and *Homo sapiens* populations, yet key early findings have largely been overlooked in later studies. Here, we present the results of new examinations of both the fossil and archaeological collections from Shukbah Cave, located in the Palestinian West Bank, presenting new quantitative analyses of a hominin lower first molar and associated stone tool assemblage. The hominin tooth shows clear Neanderthal affinities, making it the southernmost known fossil specimen of this population/species. The associated Middle Palaeolithic stone tool assemblage is dominated by Levallois reduction methods, including the presence of Nubian Levallois points and cores. This is the first direct association between Neanderthals and Nubian Levallois technology, demonstrating that this stone tool technology should not be considered an exclusive marker of *Homo sapiens*.

## Introduction

**Figure 1:**
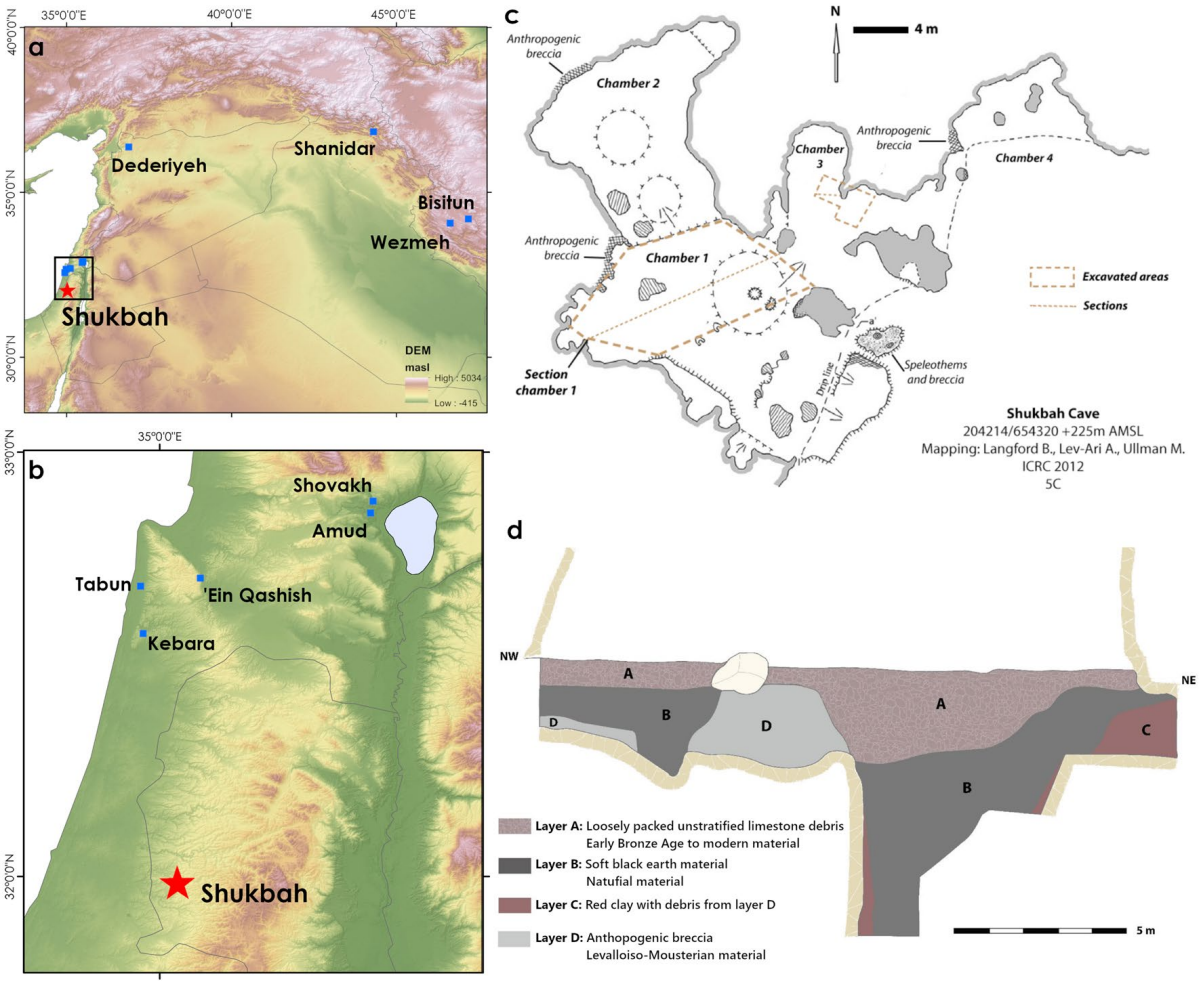
The location of Shukbah Cave, and illustration of the excavations in plan and in section. (**a**) Regional map of sites in South West Asia and (**b**) close up map of northern Israeli cave sites preserving fossil Neanderthal specimens, illustrating the position of Shukbah to the south of these sites; (**c**) a plan of Shukbah Cave (modified from Frumkin et al.^30^) illustrating areas of excavation, preserved deposits of anthropogenic breccias and the location of the illustrated section shown as (**d**) (redrawn from Garrod^56^).

Given genetic evidence for interbreeding between *Homo sapiens* and Neanderthal populations^1–6^, constraining when and where they may have encountered one another has broad ramifications for understanding our shared heritage. With a wealth of chronometrically dated Palaeolithic sites concentrated in a relatively small area, a number of which preserve fossil hominin specimens, the Levant is a key region of focus to examine biological and behavioural differences between these populations, as well as possible interactions between them. Early evidence for either populations is sparse, with Lower Palaeolithic occupations at sites such as Qesem^7,8^ reflecting broad variability amongst Middle Pleistocene *Homo* populations, and the presence of *Homo sapiens* in the eastern Mediterranean in the late Middle Pleistocene hinted at from isolated examples^9,10^. In the Late Pleistocene, *Homo sapiens* occupied the Levant during Marine Isotope Stage 5 (MIS 5: 130–71 ka)^11–13^ then are next documented in the region from ~ 50 ka onwards^14,15^. With the onset of cooler conditions at the start of MIS 4 (71–59 ka)^16–19^ fossils of Neanderthal populations have consistently been recovered from the wooded landscapes of the eastern Mediterranean coast associated with late Middle Palaeolithic assemblages^20–22^ (Fig. 1). Earlier evidence for the Neanderthal occupations of the region remain mired in controversy, such as the dating and provenance of Tabun C1^23,24^. Following MIS 5, therefore, a substantive change in hominin demography can be observed in the Levant, with an expansion of Neanderthals from their northern distribution into regions previously occupied by *Homo sapiens*. This has been interpreted as a population overturn associated with replacement of stone tools assemblages focusing on centripetal Levallois flake reduction (closely connected with *Homo sapiens*^25,26^), by those in which unidirectional convergent Levallois point production appears particularly prominent (associated with Neanderthals^27^). This apparent turnover in hominin demography and behaviour appears contemporaneous with wider changes across the landscape associated with the return of colder conditions, such as in faunal records from the Levant that document the appearance of new Palearctic taxa^28,29^, and may provide a key context for interactions between hominin populations.

Documenting and characterising the southward expansion of Neanderthal population at the onset of the last glacial phase is complicated by the scarcity of sites with suitable fossil preservation. This is compounded by the difficulties in drawing direct parallels between patterns in biological and cultural records, and historic practices of research that first documented the appearance of Neanderthals in the Levant. Across the region, sites with anthropogenic breccias have been a key source of hominin fossils, with the potential for making new discoveries at sites such as Tinshemet Cave, which have seen minimal historic intervention^30^, unfortunately rare.

Shukbah Cave (Fig. 1), located in the Judean/Hebron Hills of the Palestinian West Bank to the north of Jerusalem, is an example of a cave site that preserved an anthropogenic breccia and subject to extensive excavations in 1928 by Dorothy Garrod^31^ (SI1). These excavations yielded a rich collection of Middle Palaeolithic artefacts, fossil fauna and sparse hominin specimens (SI2) but are largely overlooked in wider studies of the region, in part due to the lack of recent analysis. Though attributed to Neanderthals, the key hominin fossils from the site were housed in Keith’s private collection throughout most of the last century^32^, prohibiting comparative analysis to test the claimed Neanderthal affiliation. Stone artefacts from the excavation were collected selectively and subsequently dispersed to several global institutions that had supported the excavations, with an emphasis on complete Levallois flakes and points, retouched tools and, to a lesser extent, cores, whereas fine debitage, simple flakes and broken pieces appear to have been discarded^33^. The first detailed assessment of the known artefact collections suggested stone technologies that are comparable with other Middle Palaeolithic sites with Neanderthal remains^33^. In particular, emphasis is placed on the prominence of unidirectional convergent Levallois point reduction, but the illustration of a Levallois point core with a distinctive distal divergent scar pattern on the flaking surface creating a medial-distal ridge is consistent with Nubian Levallois technologies^34,35^ (see SI3), hinting at evidence of greater diversity within the assemblage.

In southern Arabia, the presence of Nubian Levallois technology has been argued to reflect to expansions of *Homo sapiens* from Africa^35,36^. Connections between such technologies and *Homo sapiens* have also been proposed for the southern Levant^37,38^. Here, we re-examine fossil and archaeological finds from Shukbah Cave, exploring demographic and behavioural diversity in the Middle Palaeolithic of South West Asia. We conduct comparative quantitative analyses of the external and internal structure of the hominin mandibular molar (NHMUK PA EM 3869; abbreviated below as EM 3869) (SI4–6) and Middle Palaeolithic stone tool assemblages that were recovered from the intact, anthropogenic breccias of Shukbah Layer D (SI7).

## Results

### Non-metric traits of the EM 3869 specimen

**Figure 2:**
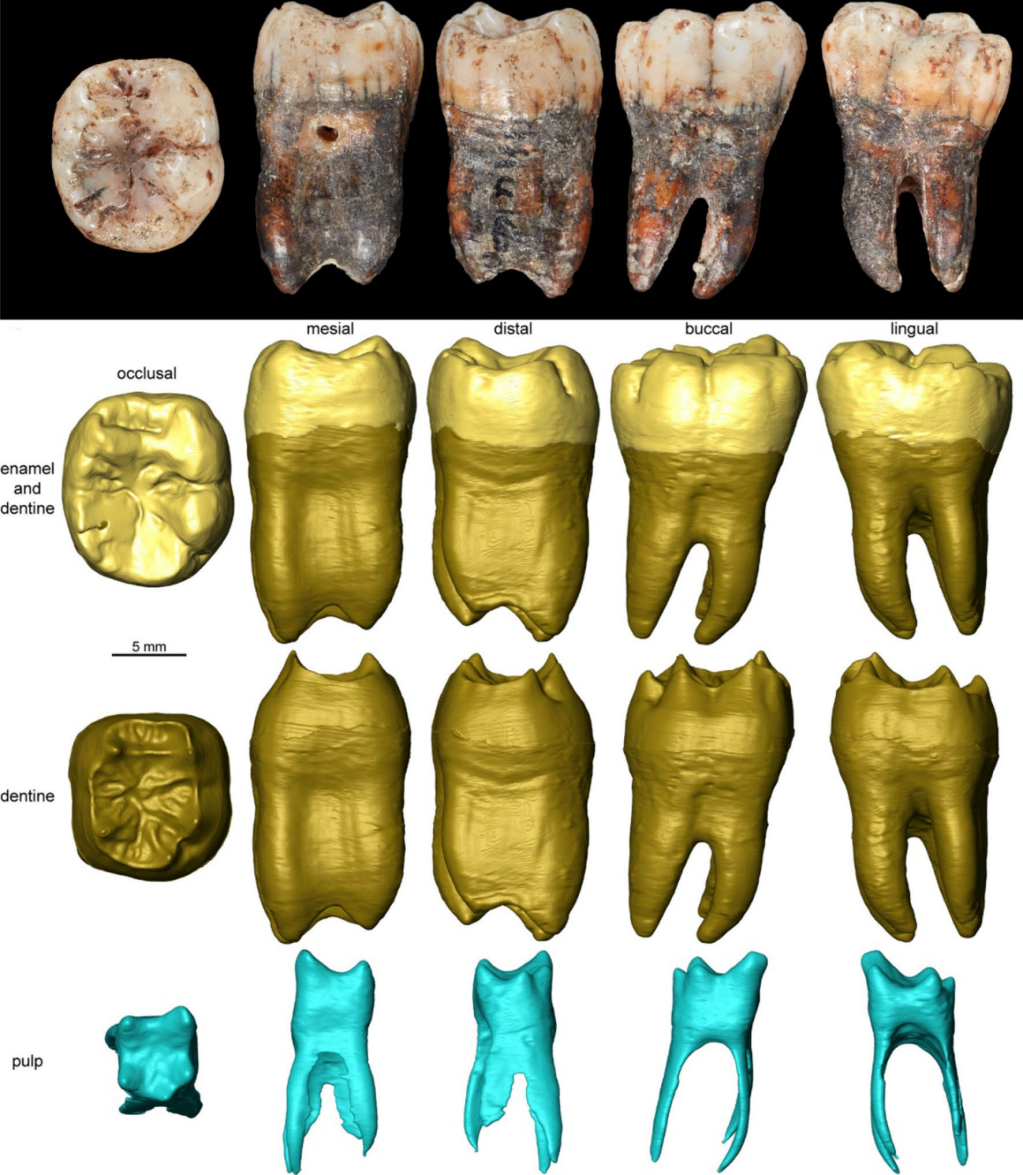
Photographic and Virtual rendering of EM 3869. Photographic and Microtomographic-based 3D surfaces of the external morphology, dentine and pulp are illustrated in occlusal, mesial, distal, buccal and lingual views.

The Shukbah tooth (EM 3869) is an isolated fully developed lower right permanent molar (Fig. 2), complete apart from a postmortem chip on the occlusal surface of the distolingual cusp, described in detail in SI4, with the key external and internal features outlined below. Starting with the external description, wear is mild, with pinpoint dentine exposure on the entoconid (wear stage 3^39^), and there is no distal interproximal facet, meaning that any tooth distal to it had not fully erupted. The regular occlusal morphology, the lack of tapering in the crown shape viewed distally, and the widely spaced and near vertical roots, suggest that EM 3869 is either an M_1_ or M_2_. The specimen presents several external (at the outer enamel surface [OES] level, and on the roots) and internal features (on the enamel-dentine junction [EDJ]) that are characteristic of Neanderthals including:

(i) the lingual convexity and buccodistal expansion of the occlusal OES outline, in contrast with a straighter lingual margin and buccodistal reduction found in modern humans^40,41^;
(ii) the presence of a wide anterior fovea and a prominent crest linking the mesial cusps (mid-trigonid crest). The mid-trigonid crest, in particular, is rare or absent in comparative groups for both M_1_ and M_2_ (SI Table 1)^42^;
(iii) the near rectangular shape of the roots, with little tapering, unlike modern humans, along with a bifurcated mesial root, and buccal and lingual marginal ridges, placed mesially and distally on the mesial root, and mesially on the distal root^43^.

**Figure 3:**
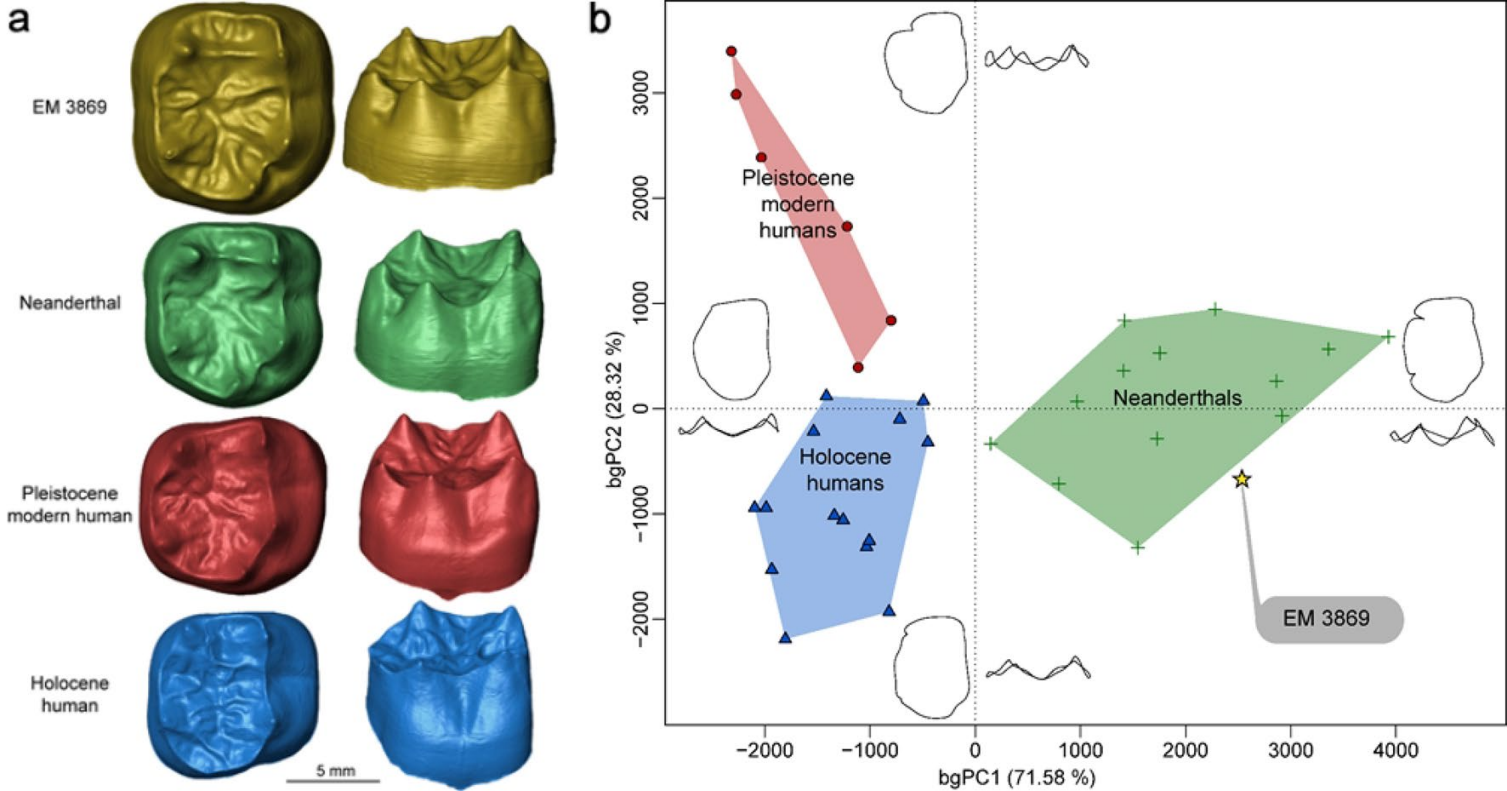
Virtual rendering of and Geometric morphometric analyses of the enamel-dentine junction (EDJ) shape of EM 3869. (**a**) Enamel-dentine junction (EDJ) of EM 3869 compared with those of Neanderthal, Pleistocene and Holocene modern human individuals. The specimens are oriented in occlusal (left) and buccal views (right). (**b**) Between group principal component analysis of the 3D semilandmark Procrustes-registered shape coordinates of the EDJ. The wireframes at the end of the axes illustrate the extreme morphological variation trends in occlusal (mesial aspect upward and buccal on the right) and buccal views (mesial aspect rightward).

The morphology of the enamel-dentine junction (EDJ) is consistent with the observations of the outer enamel surface, including the presence of a well-developed C5 dentine horn, a deep and elongated anterior fovea and a continuous mid-trigonid crest (SI Table 1 and Fig. 2). EM 3869 also shows an internally tilted metaconid dentine horn, as often seen in Neanderthals^44^, whereas it is more external and vertical in modern humans (Fig. 3a). The relatively short roots and their shape suggest that EM 3869 is an M_1_. The roots of the Shukbah tooth are similar to those of Western Asian Neanderthal M_1_, whilst the M_2_ roots are generally longer than the M_1_, less well spaced, and more distally inclined (Tabun I^45^, Amud 1^46^, Shanidar 1 and 2^47^, Kebara II^48^). We ran a 3D geometric morphometric analysis (3D GMA) of the EDJ shape and tested whether EM 3869 is more likely a M_1_ or a M_2_ and the results confirm the previous observations, the specimen being statistically attributed to a M_1_ (see “Methods”). The presence of an 'X' groove pattern (protoconid and entoconid in contact) at the OES level is both notable and unusual in an M_1_, a 'Y' groove pattern (metaconid and hypoconid in contact) being near ubiquitous in all the comparative samples (SI Table 1). However, Martinón-Torres and colleagues^49^ found only 82% with a 'Y' groove pattern in their M_1_ Neanderthal sample from largely different sites. At the EDJ level, the morphology of the trigonid crest pattern (type 10) is found in moderate frequency in the M_1_ of Sima de los Huesos hominins (21.42%) and Neanderthals (31.25%), but less frequently in modern human M_1_ (8.33%)^50^. Overall, EM 3869 exhibits a large part of the morphologically typical Neanderthal features.

### Crown dimensions of EM 3869

**Figure 4:**
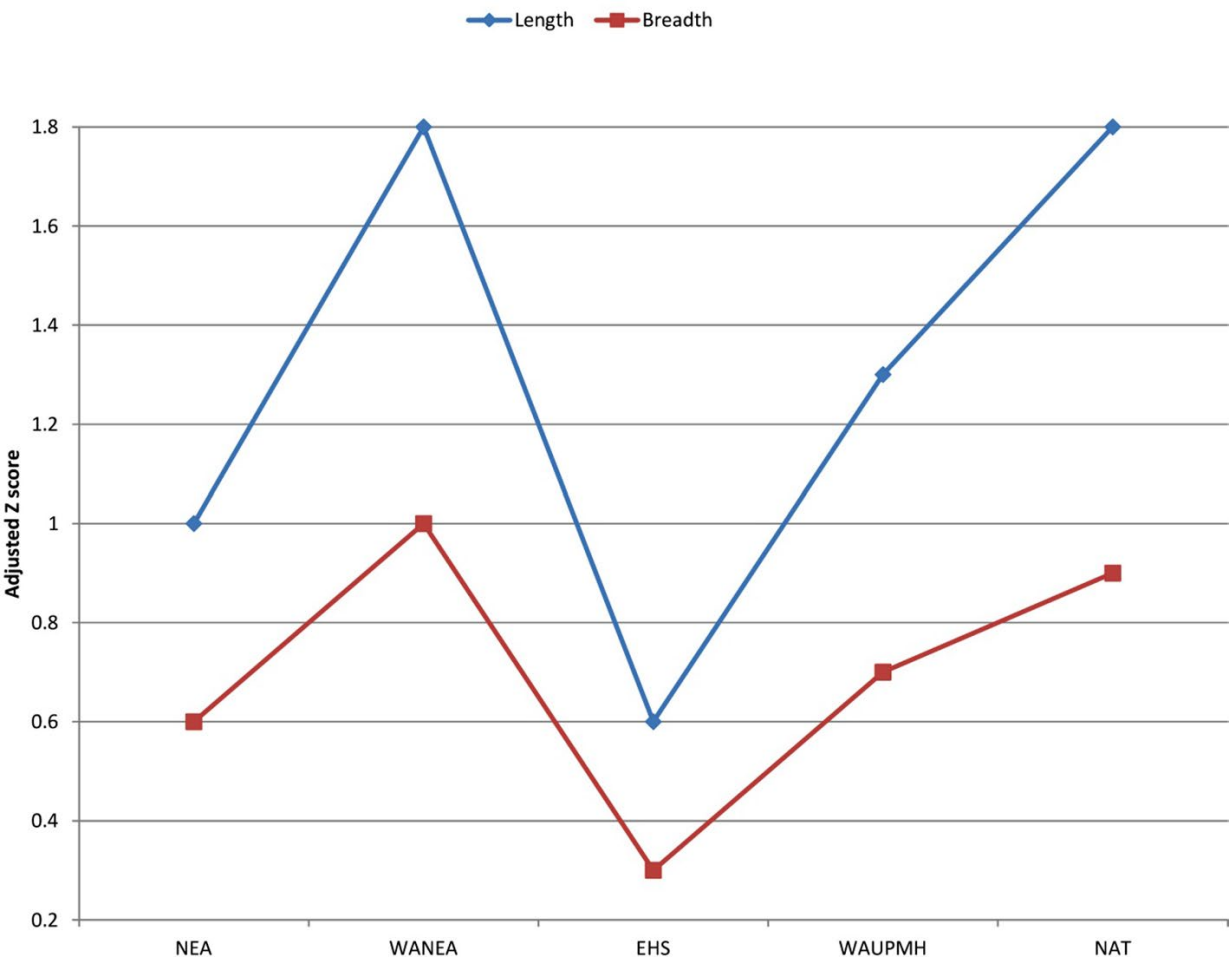
Adjusted Z-scores of the crown dimensions of EM 3869 compared with Pleistocene and Holocene hominins. *NEA* Neanderthals, *WANEA* Western Asian Neanderthals, *EHS* early *Homo sapiens*, *WAUPMH* Western Asia Upper Palaeolithic modern humans, *NAT* Natufians.

The size of EM 3869 is particularly remarkable, especially in the mesiodistal length dimension, the adjusted Z-scores for length in all the comparative groups, other than early *Homo sapiens*, being at 1.0 or above (indicating that the Shukbah specimen is statistically at or outside 95.0% of their variability; Fig. 4; SI Tables 2–6). It is only in the early *Homo sapiens* group that teeth with a greater length are found; at Jebel Irhoud^51^ and in Aterian sites^52^. Within Neanderthals, teeth of a similar length are found at Krapina^53^. The relatively greater length gives EM 3869 a narrower rectangular appearance and a crown index (breadth divided by length × 100) of 88 that is below the range for other Western Asian Neanderthals (91–101). However, many of the teeth from the Western Asian sites are worn, which will have reduced length and increased the figure for crown index. As a subset of Neanderthals, Western Asian Neanderthal M_1_ have smaller mean length and breadth dimensions, and the adjusted Z-score of EM 3869 compared with this group for the breadth dimension is at 1.0, in addition to that for the length dimension being at 1.8.

Since there are large occlusal wear facets and the entoconid dentine horn tip of EM 3869 is exposed, it suggests that a non-negligible portion of cuspal enamel has been removed by wear. In order to assess tissue proportions, we limited our measurements to the lateral portion of the crown. Lateral enamel thickness estimates are close in Neanderthals and modern humans, the former showing lower average values than the latter (SI Table 7). The Shukbah molar estimates are closer to those of Neanderthals, but also compatible with the range of modern humans (SI Table 7).

### Enamel-dentine junction (EDJ) shape of EM 3869

The three-dimensional geometric morphometric analysis (3D GMA) of the M1 EDJ conformation distinguishes between Neanderthals, Pleistocene *Homo sapiens*, and Holocene *Homo sapiens* (Fig. 3b, SI Fig. 1 and SI Table 8). The Neanderthal EDJ has a higher topography and more centrally placed dentine horn tips than in modern humans. The specimen EM 3869 falls close to the Neanderthal range, also exhibiting high and internally tilted dentine horns (Fig. 3). Results of the cross-validated bgPCA confirm a high-level of correct classification in the comparative groups and EM 3869 is classified as a Neanderthal (SI Tables 9 and 10).

### Root proportions and taurodontism in EM 3869

The roots of EM 3869 are relatively short, display four branches, two mesial and two distal, fused along most of their length. Root length in EM 3869 is a little below the mean for comparative groups of Neanderthals, fossil *Homo sapiens* and recent *Homo sapiens* (SI Table 11). The stem root portion goes lower on the buccal aspect (near mid-root length) than on the lingual aspect (only a third of root length). EM 3869 exhibits mild taurodontism (vertical expansion of the pulp chamber into the roots). Taurodontism in other Western Asian Neanderthal M_1_ is either absent (Shanidar^47^; Kebara II^48^) or mild (Teshik-Tash^54^; Tabun 1^45^; Amud 1^46^). Whilst being associated with Neanderthals, taurodontism also occurs in early *Homo sapiens* teeth from Skhūl^45^ and from Qafzeh, Jebel Irhoud, and Aterian sites^55^. Turning to the internal structure of the specimen, the pulp chamber of EM 3869 shows five well-marked horns corresponding to the main cusps. It is large, expanding low in the root until the bifurcation, before separating into four mesiodistally flattened canals, one in each root branch (Fig. 2). These observations support external assessment for some degree of taurodontism. We estimated the volume proportions between the stem and branch root portions in the Shukbah molar and it is statistically comparable with the values observed in Neanderthals but differs from fossil and recent modern humans (SI Table 12).

### The stone tool assemblage

We analysed 707 lithic artefacts from Shukbah D, constituting 57% of the total collection selected and reported by Garrod^56^ and 61% of the collection where its present location is currently known (SI7; SI Table 13), all of which are produced on varying cherts. The results of this analysis support the broad conclusion of Callander’s analysis^33^ that an emphasis on Levallois point production is evident, but our analysis reveals greater variability within the assemblage than previously acknowledged. Here, we highlight this diversity with respect to patterns of shaping flaking surfaces evident among both the core and flake populations.

**Figure 5:**
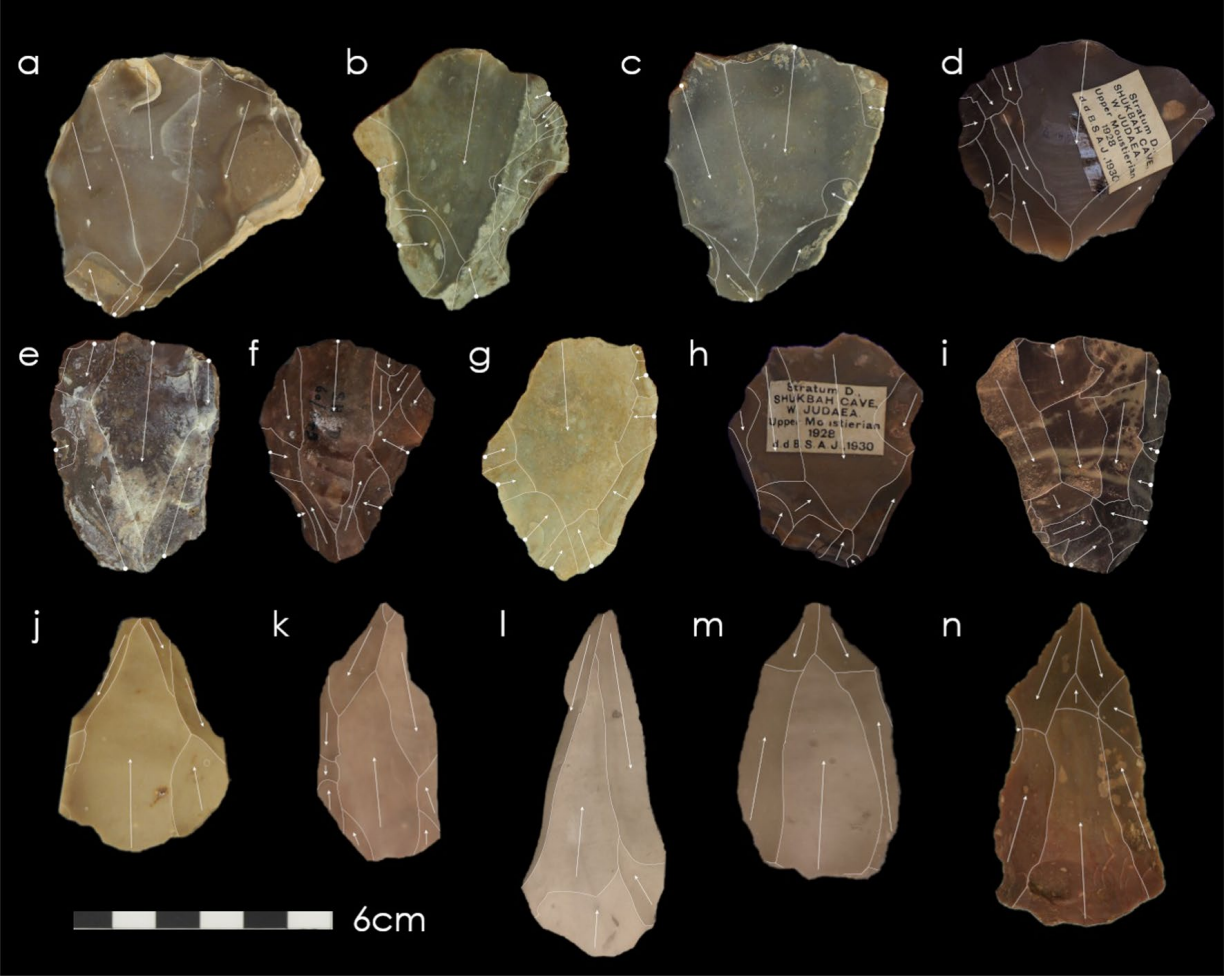
Nubian Levallois cores from Shukbah Cave (**a**–**h**) and Bisitun (**i**) and Nubian Levallois points from Shukbah cave (**j**–**n**) which feature a medial distal ridge through distal divergent and/or bilateral shaping. (**a**–**c**, **e**–**g**, **j**–**m**) Photos © UCL, Institute of Archaeology; (**d**, **h**, **n**) courtesy of the Pitt Rivers Museum, University of Oxford; i courtesy of the Penn Museum, University of Pennsylvania).

Our evaluation of the Shukbah assemblage revealed the presence of 12 Nubian Levallois points and 16 Nubian Levallois point cores (Fig. 5). Nubian Levallois artefacts are more numerous at Shukbah than at other sites where they are identified in the Levant and at many sites in Africa^34^, although not as frequent as at some sites in southern Arabia^35,57^. A more exhaustive artefact collection strategy could have further increased their frequency within the Shukbah assemblage. An attribute analysis was conducted to examine morphological differences between Nubian Levallois points and point cores and the wider evidence of Levallois point reduction strategies at Shukbah (see “Methods” and SI7).

**Figure 6:**
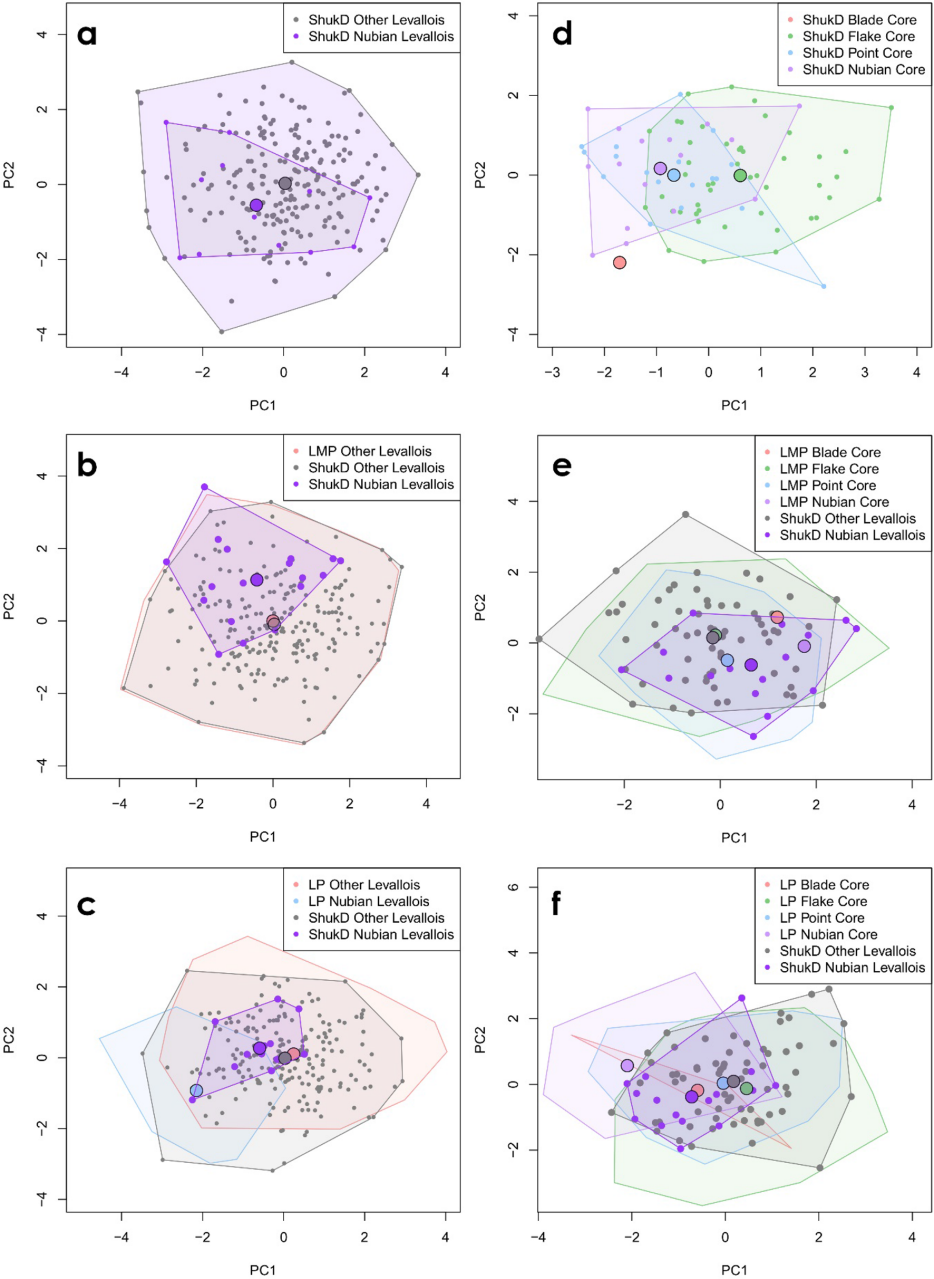
PCA biplots of from comparison of Nubian Levallois points and other Levallois Points (left), and Nubian Levallois cores and other Levallois cores (right): (**a**, **d**) Nubian Levallois points and cores from Shukbah fall within variability observed across the wider body of Levallois points and cores; (**b**, **e**) the Shukbah point and core assemblage show considerable comparability to other late Middle Palaeolithic (LMP) datasets (dated to MIS 4/3 and/or associated with Neanderthals), including Nubian Levallois specimens which fall within the range of morphological variability observed. (**c**, **f**) the Shukbah point and core assemblage, including Nubian Levallois points and cores, show considerable comparability to other Late Pleistocene (MIS 5–3) Middle Palaeolithic Levallois Points and Cores, whereas Nubian Levallois points and cores from other Late Pleistocene Middle Palaeolithic sites typically exhibit distinct differences in artefact morphology.

Multivariate analysis of the Levallois point dataset indicates that Nubian Levallois points from Shukbah D are not distinct in their overall morphology from the wider body of Levallois points at the site (Fig. 6a; SI Fig. 2). We extend this analysis and place Shukbah D within its wider context by comparing Levallois point technologies from the site to several key late Middle Palaeolithic comparative assemblages, which date to the timeframe of Neanderthal occupations of South West Asia and/or preserve Neanderthal fossils (SI7). Multivariate analysis of the dataset indicates some inter-assemblage variability amongst these sites, with the notably large collection of Levallois points from Shukbah showing no distinct difference from the total pattern of regional variability (Fig. 6c; SI Fig. 4). This suggests that selective curation has not skewed the dataset substantially. The results indicate that Nubian Levallois points from Shukbah exhibit morphologies comparable to Levallois points evident across a number of sites dating to MIS 4 and 3 and/or preserving Neanderthal fossils. Comparisons with southern Arabian assemblages that are notably rich in Nubian Levallois points (123b^58^; TH383^36^) as well as Middle Palaeolithic assemblages directly associated with other *Homo sapiens* (Qafzeh^13^; Skhul^59^) indicate significant differences between Arabian Nubian Levallois points and other Levallois points in the sample (Fig. 6e; SI Fig. 7), which are predominantly explicable through differences in size. The Nubian Levallois points from Shukbah appear morphologically similar to the broader sample of Levallois Points studied, with a subset also overlapping with variability in Nubian Levallois rich assemblages (SI Fig. 8).

Multivariate examination of the core dataset reveals substantive differences between Levallois flake and point cores from Shukbah (SI Fig. 3). The variability of Nubian Levallois cores largely overlaps with the variability observed amongst other point cores (Fig. 6b). Levallois point cores and Nubian Levallois points cores from Shukbah D exhibit comparable variation to point cores present within other later Middle Palaeolithic comparative assemblages (SI Fig. 5), further supporting the potential utility of the assemblage for comparative study. Notably, our inspection of Levallois cores from Bisitun, an undated but excavated cave site in Iran which has yielded a Neanderthal fossil^60,61^, also revealed a Levallois core (Fig. 5i) which matches the description of Nubian Levallois technology^35^, hinting at their more widespread appearance across the region. Further examination of the debitage collections from this may further support this discovery, though in many cases Nubian Levallois technology occurs in low frequency when present^34^. Examination of variability across all Levallois cores sampled from late Middle Palaeolithic comparative sites demonstrates that point cores typically exhibit a subset of variability observed among flake cores, supporting the trend identified at Shukbah (Fig. 6d; SI Fig. 6).

We examined variability among Levallois cores across a range of Late Pleistocene comparative assemblages spanning eastern Africa and South West Asia. Multivariate analysis of this dataset further supports the suggestion that Levallois flake cores are morphologically more diverse than point cores, which show a subset of this diversity, but Nubian Levallois point cores derived from southern Arabian sites demonstrate a departure from this pattern that are predominately driven by differences in core size (Fig. 6f; SI Fig. 9). Within this context, Nubian Levallois point cores from Shukbah share a common set of characteristics with other Levallois point cores from across the comparative dataset, and extending the variability observed for Nubian Levallois point cores (SI Fig. 10).

## Discussion

Our new analyses of fossil and stone tool collections from Shukbah Cave have illuminated unanticipated diversity in both Neanderthal morphology and material culture. Our study provides a thorough evaluation of the EM 3869 molar that enables a robust attribution to a Neanderthal population, corroborating earlier assessment. Based on the degree of wear, the fully developed roots and the lack of a distal interproximal facet, age at death would have been ~ 7–12 years (see SI4–SI5), with wide differences in Neanderthal dental development precluding a more refined age estimate. The size of the Shukbah molar largely exceeds that of other Western Asian Neanderthal specimens from Amud, Dederiyeh, Kebara, Shanidar, Tabun and Teshik-Tash, but more closely approximates the larger crowned European Neanderthals, and in particular those from Krapina (SI Table 3). Indeed, EM 3869 represents one of the largest M1 recovered for this taxon, adding to the morphometric variation amongst Neanderthals. Our firm attribution of EM 3869 as a Neanderthal marks the southernmost fossil of this population known to date.

The stone tool technology from Shukbah D shares the broad characteristics of other later Middle Palaeolithic assemblages found across the Levant, frequently in association with Neanderthal fossils^27,62^. However, our detailed analysis of the Shukbah lithic assemblage has revealed the presence of the Nubian Levallois method, marking the first time this technology has been found associated with Neanderthals. Considerable controversy exists regarding Nubian Levallois technology and its prominent attribution to *Homo sapiens*^34,63^. The wide distribution of Nubian Levallois technologies across time, space, and technological contexts, as well as typically appearing in low numbers, questions their utility as a robust indicator for cultural inheritance^34,63^. The appearance of Nubian Levallois technologies at Shukbah associated with Neanderthals, and potentially at Bisitun, is most simply explained by technological convergence resulting from a focus on Levallois point production. This is consistent with the broad overlaps seen between Nubian Levallois points and cores and other Levallois point production approaches. Although cultural inheritance of the Nubian Levallois method among Middle Palaeolithic/Middle Stone Age populations cannot be precluded by this finding, they remain to be demonstrated and do not represent the most parsimonious explanation. Our results indicate that any direct link between Nubian Levallois technology and *Homo sapiens* can no longer be assumed.

The Neanderthal morphology of EM 3869 and the later Middle Palaeolithic character of the stone tool assemblage from Shukbah D are consistent with wider Neanderthal occupations of the Levant between ca. 70–50 ka. Indeed, although Nubian Levallois technology has prominently been associated with MIS 5 by proponents of the ‘Nubian Complex’, a detailed evaluation indicates their recurrent appearance amongst Middle Palaeolithic and Middle Stone Age assemblages across the later Middle Pleistocene and Late Pleistocene^34^. As a result, the appearance of Nubian Levallois technology at Shukbah alone offers no clear chronological constraint, and rather the broader character of the stone tool assemblage remains comparable to other assemblages associated with Neanderthals. This is also consistent with the evaluation of the faunal record by Bate^56^, indicating the presence of voles (*Cricetinae*) and the absence of Afro-Arabian fauna, that is indicative of the wider faunal changes identified at the onset of glacial conditions in the Levant^28,29^. Combined, these support attribution of the occupation of Shukbah D to the wider timeframe of Neanderthal inhabitation of the Levant ca. 70–50 ka, though confirmation of this demands direct chronometric dating.

Examining the limits of the Neanderthal range is critical to evaluate the environmental tolerances of these populations, as well as to explore their ecological adaptability and behavioural flexibility. Our confirmation of Neanderthal occupation at Shukbah presents a notable southward expansion of the range of this population that is associated with diverse technological practices, evident in the presence of the Nubian Levallois reduction method amongst other Levallois approaches. These findings help support longstanding assumption of Neanderthal occupations at sites even further south, such as at Tor Faraj and Tor Sabiha^64^. The Neanderthal occupation of the Levant is traditionally associated with Mediterranean woodland habitats, where basecamps appear situated within rough terrain and potentially compress resource niches^22^, but occupation in seemingly arid regions of Syria^65^ and Iran^66^ suggest wider behavioural flexibility in adapting to South West Asian ecologies. Shukbah presently marks the southernmost point of the Neanderthal range, highlighting the potential importance of this region of the Levant for examining interactions with modern human populations. However, the site may have provided a vital stepping-stone for Neanderthals to expand further south into the arid landscapes beyond.

## Methods

### NHMUK PA EM 3869

The dental material available for study consists of an isolated fully developed lower right permanent molar in the keeping of the Natural History Museum, London, accession number NHMUK PA EM 3869 (abbreviated elsewhere as EM 3869). It was discovered in the Mousterian layer C/D of Shukbah Cave in Palestine during excavations in 1928^31^.

### External morphology

Most of the morphological traits studied are in the Arizona State University Dental Anthropology System (ASUDAS), and their scoring system has been followed using their reference plaques^67–69^. The degree of taurodontism of the molar is described using the volumetric bifurcation index^70^, determined from the CT-scan. Comparative data were taken from the literature; and also from direct observation of original Aurignacian material from the sites of Kebara and El Wad in the collection of the Natural History Museum, London^32^, first described by McCown and Keith^45^; identified in the relevant table legends and described in detail in SI Tables 4–6. Measurements of the Shukbah tooth and the Aurignacian material were taken with sliding callipers to 0.1 mm on the original specimens. The crown dimensions and root length were measured using the method of Moorrees^71^: crown = maximum dimensions in line with and at right angles to the buccal surface; root = maximum dimension from the cervix of the mesial/only root on the buccal side (SI Tables 2 and 11). Root stem length was measured as the distance between the cervix and the root furcation buccally. Cervical dimensions were measured as the maximum dimensions at right angles to the mesial and buccal surfaces. The mesiodistal crown dimension of the Shukbah tooth was adjusted for wear using the method of Wood and Abbott^72^. The adjusted length was used to calculate crown area and crown index, and in making comparisons with other teeth.

An adjusted Z-score method, using Student's t inverse distribution^73^, was employed to compare each of the Shukbah measurements (external, tissue proportions and volumetric bifurcation index) with the means and standard deviations of comparative groups. The formula applied was:

$$\frac{\text{(Shukbah dimension - X)}} {({\text{Invt}}_{{0.{975};n - {1}}} * \, \surd \, \left( {{\text{SD}}^{{2}} * \, \left[ {{1 } + { 1}/n} \right]} \right))}$$

where X, SD and *n* represent the mean, sample standard deviation and sample size, respectively, of the comparative sample. The interval between − 1 and + 1 comprises 95% of the variation in the comparative sample.

The level of occlusal wear was quantified using Murphy's method, as summarized by Smith^39^. All measurements and observations on the Shukbah tooth were repeated by the same observer after an interval of a month.

### X-ray microtomography

The specimen EM 3869 was scanned using the X-ray microfocus instrument (X-µCT) Nikon Metrology HMX ST 225 set at the Natural History Museum of London. Acquisitions were performed according to the following parameters: 183 kV, 195 µA, 3142 images taken over 360° (0.12° of angular step) and 0.7 s exposure time for each projection. The final volumes were reconstructed with a voxel size of 12.75 µm. The microtomographic acquisitions of the comparative fossil and extant hominid specimens were performed using various equipment including X-µCT and synchrotron radiation (SRX-µCT) and reconstructed with voxel sizes ranging from 10 to 42 µm.

### Data processing

A semi-automatic threshold-based segmentation was carried out in Avizo 8.0 (FEI Visualization Sciences Group) following the half-maximum height method (HMH^74^) and the region of interest thresholding protocol (ROI-Tb^75^), taking repeated measurements on different slices of the virtual stack^76^. A volumetric reconstruction was then generated (Fig. 2).

### 3D lateral crown tissue proportions

Because of the moderate wear affecting EM 3869, the 3D assessment of tissue proportions was limited to the lateral (non-occlusal) crown portion. A plane parallel to the cervical best-fit plane, and located between the last plane showing a complete ring of enamel and the lowest points of enamel, was used to cut the crown at the cervix. A parallel plane was set at the last point of enamel in the occlusal basin and all material above it (including the part of the crown with the cusps) was removed. The enamel and dentine portions between these two planes were preserved to estimate the lateral crown tissue proportions^77–81^. The following parameters were then calculated: the lateral average enamel thickness (3D LAET, in mm) and the scale-free 3D lateral relative enamel thickness (3D LRET)^77–81^ (SI Table 7). Adjusted Z-score analyses^73,82^ were performed on the two tooth crown tissue proportions parameters for EM 3869 in comparison with the fossil and recent hominin specimens/samples presented by Martín-Francés and colleagues^81^ (SI Table 7).

### Geometric morphometric analyses

Three-dimensional geometric morphometric analyses (3D GMA) were conducted on the virtual rendering of EM 3869 EDJ compared with Pleistocene and Holocene hominin groups. In order to assess the metameric position of EM 3869, a first analysis was run on a sample of M_1_ (SI Table 8) and M_2_ (including eight Neanderthals from Krapina in Croatia [KRD1, KRD6, KRD10, KRD80, KRD86, KRD107], Regourdou in France [Regourdou 1] and Fossellone in Italy [Fossellone 3B])^83–86^. Five semilandmark curves were set along the marginal outline of the EDJ occlusal basin between each pair or cusps, for a total of 116 semilandmarks. To assess the metameric position, we ran a principal component analysis (PCA), followed by a between-group principal component analyses (bgPCA) based on the Procrustes residuals using two groups (M_1_ and M_2_), as well as a canonical variate analysis (CVA) based on the 13 first principal components explaining ~ 90% of the total variance. Both results of the bgPCA (correct classification: M_1_ = 89%, M_2_ = 72%; posterior probability for EM 3869: M_1_ > M_2_) and CVA (correct classification: M_1_ = 91%, M_2_ = 83%; posterior probability for EM 3869: 93% M_1_) predict EM 3869 is a M_1_. To assess the taxonomic affinities of the Shukbah specimen, we then conducted 3D GMA restricted to the M_1_ sample. We computed PCA, followed by a bgPCA based on the Procrustes residuals with the following three groups: Neanderthals, Pleistocene modern humans, and Holocene humans (Fig. 3b and SI Fig. 1). The bgPCA plot group distribution was verified by computing a cross-validated bgPCA (CV bgPCA; SI Fig. 1 and SI Table 9) and a CVA based on the 11 first principal components explaining ~ 90% of the total variance (SI Table 10). The Shukbah specimen was then projected a posteriori in the bgPCA plot and the posterior probabilities that it belongs to any of the comparative groups were computed with bgPCA and CVA (SI Tables 9 and 10). Results of the bgPCA cross-validation exhibit high classification accuracy, with most misclassifications happening between Pleistocene and Holocene humans, while only one Neanderthal specimen is unclassified (SI Table 9). The CVA give consistent results, with a clear separation between Neanderthals, Pleistocene modern humans, and Holocene humans and the (SI Table 10). Altogether, the analyses are consistent with each other and confirm no spurious and inflated group separation occurs^87,88^. The analyses were performed using the package Morpho v.2.8^89^ for R v.4.0.2^90^. Allometry was tested using multiple regressions^91^ in which the explanatory variable is the centroid size and the dependent variables are the PC and bgPC scores. No allometry was detected in most analyses (*p*-value > 0.05), except along bgPC2, where a weak allometric signal (*p*-value < 0.03; R^2^ < 0.1) is detected. The differences between specimens thus mostly represent shape-variation.

### Volumetric bifurcation index (VBI) of the roots

We assessed the degree of root taurodontism in EM 3869 using the 3D bifurcation index^70^. The tooth was virtually bisected into its anatomical crown and root parts by using a best-fit plane at the cervix. An additional plane parallel to this cervical plane was placed at the level of the interradicular surface, dividing the root into the volume of the stem above the bifurcation (Vcervix) and the volume of the branches below the bifurcation (Vbranch). The volumetric bifurcation index (VBI, in %) is calculated as Vcervix/(Vcervix + Vbranch) × 100. We have applied this method to EM 3869 (Vcervix = 502.6 mm^3^; Vbranch = 234.2 mm^3^), resulting in a VBI value of 68.2% and compared the results with those of fossil and recent hominin specimens/samples (SI Table 12). Adjusted Z-score analyses^73,82^ were performed on the VBI value of EM 3869 in comparison with the fossil and recent hominin specimens/samples presented by Kupczik and colleagues^55^ (SI Table 12).

### Lithic analyses

Stone tool artefacts from Shukbah D included in the analyses were studied from collections held at the Institute of Archaeology (UCL), the British Museum, the Pitt Rivers Museum (University of Oxford), the Museum of Archaeology and Anthropology (University of Cambridge) and the Peabody Museum (University of Harvard). Additional stone tool assemblages included in the comparative analyses include samples from Tor Faraj and Tor Sabiha (Department of Anthropology [Tulsa]), Bisitun (University of Pennsylvania Museum of Archaeology and Anthropology), Kebara and Qafzeh (Institute of Archaeology, [HUJ]), Ksar Akil (Peabody Museum [Harvard]), Skhul (Pitt Rivers Museum, [Oxford]). Aduma and Omo (National Museum of Ethiopia, Addis Ababa), 123b and TH383 (Dhofar Archaeology Project), Al Wusta, Al Marrat Jebel Katefeh, and Mundafan (National Museum, Riyadh). Nubian Levallois points and cores have been differentiated from other Levallois point reduction approaches by the presence of a steep medial-distal ridge produced through a combination of distal divergent or lateral removals which help to guide the preferential flake removal^35^. Metric variables, including maximum, axial and platform dimensions were measured using Vernier callipers, with platform angles measured using a goniometer. Morphological indices used here include elongation (Length/Width), Proximal Shape (Proximal/Medial Width), Distal Shape (Medial/Distal Width), Flattening (Medial Width/Thickness) and Tip Cross Sectional Area (TCSA; Distal Width x Distal Thickness^92^). Categorical variables recorded include blank type, dorsal scar pattern, flaking surface scar pattern, core typology and blank typology. Attribute datasets were normalised using bestNormalize^93^, scaled and centred as part of the multivariate analysis (Principal Components Analysis) using the function prcomp (library stats), in R v.4.0.2^90^.

## Supplementary Information

Supplementary Information.

## Data Availability

The authors declare that all data supporting the findings of this study are available within the paper [and its Supplementary information files] or are available from the corresponding author on reasonable request.
