# Supplementary material for: Nubian Levallois technology associated with southernmost Neanderthals
Source: Sci Rep. 2021 Feb 15;11:2869. doi: 10.1038/s41598-021-82257-6 (PMC7884387; doi:10.1038/s41598-021-82257-6)
Supplement: Supplementary file 1 — Supplementary Information. [file 41598_2021_82257_MOESM1_ESM.docx]

**Supplementary Information:**

**Nubian Levallois technology associated with southernmost Neanderthals**

James Blinkhorn, Clément Zanolli, Tim Compton, Huw S. Groucutt, Eleanor M. Scerri, Lucile Crété, Chris Stringer, Michael D. Petraglia, Simon Blockley

**Supplementary Information S1. Excavations at Shukbah Cave**

Shukbah Cave is located in the Judean/Hebron Hills in the Palestinian West Bank, north of Jerusalem, and was discovered in 1924 by Mallon^1^ on the north bank of Wadi (Nahal) Natuf at the base of a steep cliff approximately 25m above the wadi bed, within the massive dolomite Weradim Formation^2^. The cave has one large entrance (11 m wide x 7 m high) with two smaller side entrances which join with the main chamber through side passages, all of which are connected by a rockshelter at the current cliff-face. Two open chimneys occur in the roof of the main chamber. Sediments contained within the cave derive from anthropogenic sources, as well as mixed natural sources including guano, dust and disintegrated rock, with little input of colluvial terra-rossa^2^.

Archaeological excavations in Shukbah Cave were undertaken by Garrod in a single season in 1928^3,4^, commencing in April and closing in mid-June, after which her fieldwork program was concentrated upon the cave sites of Mount Carmel^5^. A wide trench was excavated in the main chamber, with sediment depths exceeding 5m in some areas where the excavations did not reach bedrock. An additional small sounding trench was excavated in a side chamber. The sediment sequence was originally described in three levels, (A, B, C/D), prior to differentiating four levels (A to D), which are steeply inclined to the deeper part of the section. Layer A, at the top of the sequence, ranges in thickness from 0.8-3 m, is comprised of angular limestone fragments, loosely packed with brown earth^3,4^. Layer B, ranging from 0.5-3.5 m thick, is predominately composed of black hearth materials with an erosional contact with underlying sediments. Layer C is composed of eroded and redeposited material from the underlying deposit, formed predominately of abraded breccia and red clays. Layer D, ranging from 0.2-2.5 m thick, comprises a limestone breccia including well marked hearths, showing considerable degrees of consolidation in contrast to the overlying deposits but has been subject to some erosion, compaction, and subsidence.

Three layers (A, B and D) present undisturbed archaeological horizons, whereas Layer C included abraded material, likely to be derived from Layer D. Layer A represents episodes of occupation of the cave from the early Bronze Age though to the Byzantine period and more recent periods, based upon the nature of ceramics present. The archaeological collections from Layer B constitute an Upper Natufian occupation, yielding an abundant microlithic industry with animal remains associated with the black hearth deposits, alongside eleven inhumations. Layer D was reported to contain large quantities of animal bones and Middle Palaeolithic stone tools, often concentrated in the hearth deposits, alongside a small number of hominin fossils.

**Supplementary Information S2. Recovery of the Shukbah D Molar**

The molar tooth from the Mousterian level C/D was discovered on May 15th. This was in the seventh week of work in the cave, the previous weeks having been occupied by the excavation of Layer B, with its numerous human bones and burials, faunal remains, and flint and bone tools of the culture that Dorothy Garrod later defined and named (after the site) "Natufian".

Garrod recorded the first definite Mousterian discovery at Shukbah in her handwritten diary of the excavation^6^:

"(May) 15th. C continues very hard. Bones abundant near edge of pit. "Human molar found at about 2m.30 from surface, and 2m. from highest point of the lump of C."

Layer C was a hard breccia, only present in the eastern area of the cave (cf. Garrod & Bate^4^, p5, for section drawing which makes this clear, reproduced in Fig. 1). The breccia underlay the Epi-Palaeolithic Natufian Layer B, which Garrod had begun to excavate on April 5th. Layer C contained a flint industry which she recognised as Mousterian, a situation of which she became aware on this first day of digging. Later she realised that Layer C had been re-deposited from the Mousterian Layer D, which underlay the Natufian elsewhere in the cave; thus, in subsequent papers^3,4^, she usually only refers to Layer D.

The molar was in fact the second Mousterian human discovery at Shukbah. Garrod had recorded in her diary on May 12th that *"C* contains many bones" (i.e., animal), but among them:

"One fragment with glenoid fossa may be human, but the break is old & edges slightly worn."

She did not at this time attribute the fragment to any human species. Only in her first published paper^3^ does she use the term Neanderthal (quoted below), though she uses "Mousterian" in the diary in referring to the older lithic industry. She did not use 'Neanderthal' in her final report on Shukbah^4^. This is probably due to her reluctance to disagree with Sir Arthur Keith's later interpretation of the Tabun and Skhūl populations at Mt. Carmel as one variable species; "*Palaeanthropus palestinus*".

In Garrod's first brief published report (p183)^3^ she defines the flint industry of Layer D as "Upper Mousterian", which "differs markedly from the industries of the same stage in Western Europe in the greater variety of its forms and in its more delicate technique, which approaches that of the Upper Palaeolithic.... When I showed some of the Shukbah implements to the Abbe Breuil he did not hesitate to describe them as Aurignacio-Mousterians". Garrod is also now unhesitating in defining the molar tooth and the fragment of the glenoid fossa ― "Both these conformed to the Neanderthal type." *(ibid.)*

## The Shukbah molar was originally described by Sir Arthur Keith^7^, who noted its remarkable size and identified it as a Neanderthal lower right permanent second molar. Points made by Keith were:

1. The tooth dimensions exceed those of the corresponding Mauer tooth, and are similar in size and morphology to Krapina C. If its owner had reached maturity, the length of his dental palate would have been about equal to that of La Chapelle man.

2. The tooth was not a loose member that had dropped from its socket; remains of decaying alveolar bone covered its roots when first found.

3. Only a moderate degree of taurodontism is present, much less than is found at Krapina.

4. The combination of characteristics found in the tooth, both in terms of size and morphology, occur only in those of Neanderthal stock.

5. The tooth is virtually unworn, and has an impress on its anterior aspect from contact with its neighbour in front but none on its hinder aspect. It is therefore likely to be recently erupted and from a youth aged 12.

6. All five main cusps are fully and regularly formed, and the enamel on them has a sharply cut crystalline form.

Subsequently, in McCown and Keith^8^, Keith revised his opinion and described this tooth as a first molar, and noted its similarity in crown and root form to the teeth of Tabun. Possibly this revision was due to the similarity in the shape of the roots to those of the *in situ* first molars of the Tabun Mandibles I and II. This tooth was in a collection of material from the caves of Shukbah, El Wad and Kebara held by Arthur Keith, and deposited with the Natural History Museum, London, in 2001^9^.

Other material found in layers C/D that Keith^7^ considered to be Neanderthal were a fragment of temporal bone (including the glenoid fossa) (see above) and the upper part (tabular) of a frontal bone. Other human remains found in this layer were considered to be intrusive, and those of a ‘race of modern man’. In describing the temporal fragment Keith^7^considered that it was from an immature skull and may be from the same individual as the molar. He noted that the post-glenoid process was of remarkable size and strength for such a stage of immaturity and, as with the molar, could only find this combination of characteristics in Krapina C. The tabular part of a frontal bone also came from a young individual.

**Supplementary Information S3: Description of Nubian Levallois technology**

Nubian Levallois technology, and wider assemblage groupings identified as ‘Nubian’, have been tackled at length by Groucutt^10^, with salient points for this analysis revisited here. Nubian Levallois technology was first described by Seligman^11^, identifying Levallois (tortoise) cores that presented distal modifications to create a distinct ‘beak’. Guichard and Guichard^12^ present the first technical description of Nubian Levallois technology, differentiating the use of two distal removals to shape the core margins and produce a short ridge and aid the removal of a Levallois point as the Type 1 approach, and through lateral removals as the Type 2 approach. A survey of the literature concerning Nubian Levallois technology indicates broad agreement that there is not a strict dichotomy between these types, nor are they inherently discrete from other Levallois reduction approaches^10^. With this caveat in mind, the use of distal and/or later removals to form a distal medial ridge to guide the production of a convergent preferential flake (point) differentiates reduction schemes that have been categorised as Nubian Levallois from wider Levallois approaches, and especially the focus on unidirectional or unidirectional convergent flaking that is otherwise a prominent feature of Levallois point production.

Considerable debate exists regarding the significance of Nubian Levallois technology, and particularly the extent to which its use may reflect patterns of cultural transmission or convergence. The presence of Nubian Levallois technology has been used to ascribe sites to the ‘Nubian Complex’, a culture historical grouping of assemblages broadly dating to the early Late Pleistocene and first used in NE Africa^13^. Groucutt^10^ highlights that proponents of the ‘Nubian Complex’ focus upon the presence or absence of Nubian Levallois technology for group membership, rather than any proportional threshold in the frequency of occurrence, and where present, Nubian Levallois technology typically occurs in low frequency. It is only in a few sites, particularly in the Dhofar region of southern Arabian, that Nubian Levallois technology is a dominant reduction strategy in assemblages ascribed to the ‘Nubian Complex’^14^. Both the chronological and geographic distribution of sites in NE Africa and Arabia which include Nubian Levallois technology have led to suggestions that the appearance of such artefacts offer cultural *fossil directeurs* for *Homo sapiens*^14–16^*.* It is notable that a possible association between Nubian Levallois technology and fossil specimens of *Homo sapiens* only occur at a single site attributed to the ‘Nubian Complex’^17^, where the stratigraphic relationship between fossils and stone tools is complex, and the age of the human fossils (from a burial) can perhaps most reliably be dated to Marine Isotope Stage 2^10^. This, alongside the low incidence of Nubian Levallois technology in most sites where it is present and the widely acknowledged gradational differences between Nubian and other Levallois point production schemes present problematic grounds to assert the use of Nubian Levallois methods as unique to *Homo sapiens*. An alternative interpretation suggests that the appearance of Nubian Levallois reduction methods represents convergent evolution of alternate modalities of controlling flaking surfaces to produce points in Levallois reduction systems^10,18,19^. Nubian Levallois technology has been identified across a wide geographic and ecological range, spanning southern Africa^18^ to western India^20,21^, where alternate modes of Levallois reduction are also present. Convergent evolution offers a simpler explanation for the appearance of Nubian Levallois technology across this vast range than either its production by a single population or its dissemination via a direct line of cultural transmission. Put simply, any association between *Homo sapiens* and Nubian Levallois technology remains to be demonstrated.

**Supplementary Information S4. Description of Shukbah isolated fully developed lower right permanent molar NHMUK PA EM 3869**

Condition: Both the crown and root of the tooth are complete and well preserved, apart from a postmortem chip on the occlusal surface of the entoconid. There are patches of concretion on the occlusal and lingual surfaces of the crown, and a black deposit on the roots. There is a drill hole on the mesial face of the root stem resulting from an unsuccessful attempt by the Max Planck Institute for Evolutionary Anthropology to abstract DNA in 2016.

Wear: There is mild wear on this tooth (grade 3) and pinpoint dentine exposure on the entoconid. There are mesial and distal phase 1 facets on the metaconid, protoconid and hypoconid, but only a single centrally placed facet on the entoconid. The phase 2 facet on the protoconid is horizontal but on the hypoconid and cusp 5 (hypoconulid) they are on the lingual slope of the cusp. The greatest wear is on the entoconid and cusp five, on which the single facets cover most of the cusp. Viewed distally the wear on these cusps is mildly concave. The least wear is on the metaconid. Overall, the degree of wear on phase 1 and phase 2 facets is similar. As seen with a 12x magnifying glass, the facets have multi-directional fine striations. There is also fine pitting on these, especially distally on the entoconid. There are vertical striations visible on the buccal and distal surfaces.

Mild regular chipping of the occlusal rim occurs mesially on the interproximal facet, buccally on cusp 5, and distally on the entoconid. There is also some chipping to the cusp tips of the hypoconid and cusp 5, and on the distal phase 1 facet on the protoconid.

There are small areas of pitting near the occlusal edge on the buccal and lingual faces of the tooth. The pitting may be non-masticatory in origin. This type of wear is not uncommon (e.g., it has been seen at the Middle Pleistocene site of Atapuerca-SH in Spain [observed by TC on a cast] and on the Le Moustier Neanderthal^22^). It possibly results from holding items against the teeth while they are being worked on.

The mesial interproximal facet measures 4.5 mm buccolingually x 2.2 mm vertically. It is mildly concave viewed occlusally and has an occlusal tilt. The surface is heavily pitted but no subvertical grooves are apparent. There is no distal interproximal facet.

Pathology Caries is absent, as also are hypercementosis and calculus. There is a distinct hypoplastic line (furrow hypoplasia) on the crown at 1.5 mm from the cervix, three faint lines above this up to 3.5 mm from the cervix, on the buccal surface, and one faint line at 2.5 mm above the cervix on the mesial surface (seen with a 12x magnifying glass). Enamel defects of this nature are considered to be related to developmental stress^23^. Using modern human development times^24^ age of occurrence would be ~1.5–3 years, but ~1.3–2.5 years using development times found in some Neanderthal teeth^25,26^. Overall, where not obscured, the perikymata are irregular. There is no evidence of malocclusion.

Morphology Crown morphological characteristics of the tooth are summarised, along with comparative data, in Supplementary Table 1. See section SI6 for a description of the traits referred to below. The occlusal surface has a rounded rectangular shape, flat mesially, notably rounded lingually, and buccodistally expanded. The buccal and lingual surfaces are both mildly convex viewed distally, but there is no cingulum and no waist or overhang of enamel at the cervix. Viewed buccally the distal surface is convex and the mesial surface is flared. Of the four main cusps, the protoconid is the largest and the hypoconid the smallest. There is an 'X' groove pattern, a buccally placed grade 4 cusp 5 (hypoconulid), and a small grade 1 cusp 7 (interconulid). The presence of cusp 6 (entoconulid) cannot be determined at the enamel surface due to wear but the lack of a dentine horn at the enamel dentine junction confirms its absence. The mesial margin is present as two tubercles, with three grooves going over it. There is a wide grade 4 anterior fovea and a very pronounced mid-trigonid crest (grade 1B). Distal trigonid crest, posterior fovea, protostylid and enamel extension are all absent. The presence / absence of deflecting wrinkle cannot be determined due to wear. On the occlusal surface there is a pit in the buccal occlusal groove between the protoconid and the hypoconid, and a crest across the groove between the hypoconid and cusp 5, at its junction with the distal occlusal groove. There is mild wrinkling. There are distinct metaconid mesial and distal accessory tubercles. The distal accessory tubercle (post metaconulid) is equivalent to cusp 7 grade 1A in the ASUDAS. The buccal surface grooves between the protoconid and the hypoconid, and the hypoconid and cusp 5, are both distinct, and that between the protoconid and the hypoconid reaches the cervix, with a mild crest across the midpoint. There is a mesially placed narrow vertical groove on the buccal surface of the protoconid, emanating from the occlusal margin. This corresponds to a grade 1 buccal surface vertical groove / cingular remnant. A very small enamel pearl can be seen on the buccal surface of the root.

The two roots are rectangular in shape, with convex sides and little tapering, near parallel, and well separated. The mesial sides of both roots have prominent rounded ridges running down the buccal and lingual margins. The distal side of the mesial root also has this feature, with a deep groove on the buccal side and a lingually placed vertical ridge. The distal side of the distal root is flat and has faint buccal and lingual vertical grooves. The mesial root is vertically convex, with the apex inclined distally, and the distal root is straight, inclined distally. Both roots have bifurcated apices, the longest part of the bifurcation being on the buccal side. There is mild taurodontism. Shaw^27^ does not give ranges of relative measurements for the four categories, but from his figure 3 the Shukbah molar would be described as hypotaurodont. Using Keene's method (measurements taken from X-ray photograph)^28^, the value for the Shukbah molar is 23% (cynodont, but just below the level for hypotaurodont [25–49.9%]).

**Supplementary Information S5. Age at death and sex**

Distinct differences have been found in dental development times of Neanderthals, some having development times considerably in advance of recent humans and others having development times within the range of recent humans. Work on the dental development of Neanderthals by Smith and colleagues^25,26^ suggests M_1_ eruption before the age of six and M_2_ eruption at the age of eight, this putting an upper limit on the age of the Shukbah M_1_, since there is no distal interproximal facet. Unfortunately, there were no relevant data for M_1_, but age of death for Krapina Maxilla B was determined as being 5.9 years, and there are slight wear facets on the incomplete M^1^ of this specimen (root three quarters complete). La Quina H18 maxilla is developmentally younger, but the M^1^ have greater wear (though less than the Shukbah tooth). However, the Shukbah M_1_ is fully developed, unlike the Krapina Maxilla B M^1^, with the root apices closed, and this is the case in the Scladina juvenile, for which age of death is eight years, though the age of M_1_ completion was not known^25^. In view of the mild amount of wear on the Shukbah M_1_, against the greater wear on the Scladina M_1_, where there is wider dentine exposure^26^, likely age of death is suggested as being ~7–8 years. However, in contrast to this advanced development, a juvenile Neanderthal from El Sidrón has been shown to have had a recent human pattern of development of the dentition^29^. This would give an age range of ~9.5–12 years between completion of M_1_ and eruption of M_2_^24^. Therefore, in view of the apparent variation in dental development times of Neanderthals, it is necessary to give a wide possible age range of 7–12 years. The considerable size of the tooth suggests that it came from a male.

**Supplementary Information S6. Trait descriptions**

Those traits described below that are included in the ASUDAS^30,31^ are denoted by an asterisk. Scoring is present (1)/absent (0) unless otherwise stated.

Groove (fissure) pattern* M_1–3_ Y: metaconid and hypoconid in contact; X: protoconid and entoconid in contact; +: protoconid, metaconid, entoconid and hypoconid all in contact.

Cusp 5 (hypoconulid)* M_1–3_ Presence of hypoconulid on distal occlusal aspect between entoconid and hypoconid. Scored as size grades 1–5. Position recorded; distal or buccal.

Deflecting wrinkle* M_1–3_ Median ridge on metaconid constricted (grade 1) or distally deflected (grades 2 and 3).

Mid-trigonid crest* M_1–3_^32,33^ The median ridges of the mesial cusps (protoconid and metaconid) link to form a crest across the mesial occlusal groove. Scored as grades 1A: thin sharp crest; 1B: thick rounded crest.

Distal trigonid crest* M_1–3_ A crest connecting the distal accessory ridges of the protoconid and metaconid. In this study scored as present (1) or absent (0).

Trigonid crest at the enamel dentine junction (EDJ) M_1–3_^34^ A trigonid crest (middle or distal) at the EDJ may originate from any of the three occlusal surface lobes of each of the mesial cusps (protoconid and metaconid). These are: mesial; middle or essential; and distal. Crests may also originate from the mesial marginal ridge. There are four grades of expression:

0: absent or weakly expressed ridges on either or both of the mesial cusps;

1: moderately expressed ridges on both cusps that do not join at the sagittal sulcus and are widely spaced;

2: presence of a crest whose height dips and/or is much reduced at the sagittal sulcus but remains continuous;

3: presence of a crest that remains high from cusp tip to cusp tip. There may be only a slight dip at the sagittal sulcus.

Grades 0 and 1 are classified as crest absent, and grades 2 and 3 as crest present. There is greater variation in form of trigonid crests at the EDJ than at the outer enamel surface (OES). Bailey and colleagues^34^ recorded six variants and Martínez de Pinillos and colleagues^35^ defined fourteen.

Cusp 6 (entoconulid)* M_1–3_ Presence of entoconulid on distal aspect of tooth between hypoconulid and entoconid. In this study scored as present (1) or absent (0).

Cusp 7 (interconulid)* M_1-3_ Presence of interconulid (metaconulid) on lingual aspect of tooth between metaconid and entoconid. Scored as size grades 1–4 (excluding grade 1A).

Anterior fovea* M_1–3_ A pit or buccolingually long depression on the occlusal surface between the mesial marginal ridge, and the mesial accessory ridges of the protoconid and the metaconid. Scored as size grades 1–4.

Metaconid mesial and distal accessory tubercles M_1–3_^36^ Termed as a separation of the mesial/distal accessory ridges of the cusp from the central (essential) ridge. The two normal grooves on the metaconid, mesial and distal, between the central ridge and the mesial and distal ridges, may intersect the lingual margin to form cuspules on the mesial and distal slopes of this cusp. Scored separately as present (1)/absent (0). The metaconid distal accessory tubercle is also referred to as the post-metaconulid by Grine^37^, and appears on the ASUDAS Cusp 7 dental reference plaque as grade 1A^30^.

Protostylid* M_1–3_ A paramolar cusp on the buccal surface of the protoconid, and associated with the buccal groove separating the protoconid and hypoconid. Scored as size grades 1–7. Grade 1 is a pit in the buccal groove.

Buccal surface vertical grooves / cingular remnants M_1–3_^38–40^ Vertical grooves (cingular remnants) may occur on the buccal surfaces of the protoconid, the hypoconid and cusp 5. They can be wide and well-defined, and run the whole height of the crown, or faint and narrow and, in some cases, only evident near the occlusal edge. On the protoconid and the hypoconid they may occur mesially and/or distally and may be multiple. Scoring: scored as present/absent in each of five positions, A to E (A: protoconid mesial; B: protoconid distal; C: hypoconid mesial; D: hypoconid distal; E: cusp 5 mesial) and intensity (grade 1: fine and narrow, may be at occlusal edge only; grade 2: wide and prominent, can be clearly seen viewed occlusally). Additional grooves and ridges recorded. Frequency of occurrence has been found to decrease from position A to position E, and increase from the first molar to the third molar^39^.

Enamel extensions* M^1–3^, M_1–3_ Projections of the buccal enamel border in an apical direction on premolars and molars. Scored as size grades 1–3. Trace 0.5 mm not scored.

Pearls M^1–3^, M_1–3_ Islands of enamel on premolar and molar roots.

Taurodontism M^1–3^, M_1–3_ The pulp chamber is expanded and extends vertically into the roots. Kallay^41^ identified three types of taurodontism: supraradicular, where the pulp chamber is enlarged above any furcation of the roots, and radicular and total, where there is only the single root, with the pulp cavity extending to the apex, these types being differentiated by the pulp cavity being hourglass shaped or barrel shaped respectively. The Shaw^27^ definition has ‘normal’ (cynodont) and three grades of taurodontism, namely hypo-, meso- and hypertaurodontism, which approximately equate to the body of the tooth (root stem, from cervix to upper end of groove marking the division, or partial division, of the roots) being a quarter, half or three quarters, respectively, of the total length of the root.

Shifman and Chananel^42^ used the distance between the bicervical line and the highest point on the floor of the pulp cavity of the tooth to distinguish between taurodont and non-taurodont molars, this being preferable to taking the measurement from the roof of the pulp chamber, which can vary over time, as the tooth wears, due to the deposition of secondary dentine, and proposed the following categories: non-taurodont molars (<2.5 mm); taurodont molars: hypotaurodont (2.5–3.7 mm); mesotaurodont (3.7–5.0 mm) and hypertaurodont (5.0–10.0 mm).

Keene^28^ further defines these categories in terms of a taurodontism index. This is (‘a’×100)/‘b’ where ‘a’ is the minimum vertical height of the pulp chamber, and ‘b’ is the distance between the lowest point of the roof of the pulp chamber and the apex of the longest root. The value ranges for each grade are; hypo: 25–49.9%, meso: 50–74.9% and hyper: 75–100%.

Kupczik and Hublin^43^ and Kupczik and colleagues^44^ used a volumetric bifurcation index calculated as Vcervix/(Vcervix + Vbranch) x 100, where Vcervix is the volume of the root stem above the bifurcation, and Vbranch is the volume of the roots below the bifurcation. Corresponding with the classification scheme of Keene^28^, a value of 0−24.9% denotes a cynotaurodont molar; a value of 25−49.9% a hypotaurodont molar; a value of 50−74.9% a mesotaurodont molar; and a value of 75−100% a hypertaurodont molar.

**Supplementary Information S7: Lithic Technology**

**Description of the Shukbah assemblages**

Artefact collection and curation practices from the excavations at Shukbah were selective, rather than systematic, and Garrod reports a lithic inventory of 1235 artefacts from Shukbah D^4^. This collection was divided between a number of institutions that supported Garrod’s work as well as through obligations to national institutions. Callander^5^ was able to trace 93.8% of the known Shukbah D assemblage, spread amongst ten institutions (Supplementary Table 13).

In the initial report on the Shukbah D assemblage, Garrod^3^ identified the technology as ‘Upper Mousterian’, noting the particularly delicate technique used in its production, and the presence of Upper Palaeolithic tool types. Following her excavation of further Middle Palaeolithic sites at Mount Carmel, Garrod described the Shukbah D assemblage as a 'Mousterian industry of Levallois tradition which represents the last stage of the Middle Palaeolithic in the Near East' ^45^, particularly given the nature and form of the retouched assemblage and the presence of flat, regular retouch. After nearly a century, relatively little has changed in the assessment of the place of the assemblage in the wider research landscape (e.g.^46^) and its comparability with late Middle Palaeolithic assemblages, or ‘Tabun B’ type assemblages.

The first detailed analysis of the Shukbah D assemblage was conducted by Callander^5^, who studied 657 artefacts from Shukbah D from collections held at the Institute of Archaeology (UCL), British Museum, Museum of Archaeology and Anthropology (Cambridge), Institut de Paléontologie Humaine, Paris, and the Rockefeller Museum, Jerusalem. This study employed a *Chaîne opératoire* approach to examine reduction sequences and reported a range of technological indices to enable comparisons with other assemblages studied in the latter half of the twentieth century. Within Callander’s approach, the dominant features of the reduction strategy were emphasised, highlighting the importance of unidirectional convergent core reduction resulting in an abundance of Levallois point blanks. This result was consistent with Garrod’s original appraisal of the Shukbah D assemblage as well as Copeland’s^47^ definition for Tabun-B type industries, which represent the final Middle Palaeolithic phases in the Levant. In particular, close comparisons are drawn with the stone tool assemblages from Kebara.

**Lithic Artefact Datasets**

We present the analysis of both the blank and core dataset at three levels: examining variability within the Shukbah Assemblage, examining how variability observed at Shukbah compares to other late Middle Palaeolithic assemblages from the Levant and neighbouring areas (including Bisitun, Kebara, Ksar Akil, Rosh Ein Mor, Tor Faraj, Tor Sabiha), and examining how variability observed at Shukbah compares to Late Pleistocene South West Asian Middle Palaeolithic assemblages (including late Middle Palaeolithic Levantine assemblage list above as well as TH123b, Qafzeh and TH383).

We have inspected lithic collections from Shukbah D at the Institute of Archaeology (UCL)(n=530), British Museum (n=31), Museum of Archaeology and Anthropology (Cambridge)(n=60), Pitt Rivers Museum (Oxford)(n=37), and the Peabody Museum (Harvard)(n=48). In addition, the whereabouts of Shukbah D specimens were unknown at the Manchester University Museum on initial enquiry (2018). An additional 37 artefacts were therefore available in the largest collection at UCL, potentially reducing the number of artefacts with unknown locations. A total of 707 artefacts were available for examination, which included 100 cores, 429 unretouched flakes, 177 retouched flakes and one flaked piece, all of which are produced on chert. Here, we focus on Levallois cores and blanks, and differentiate between flakes, blades (with a length: width ratio >2) and points (with convergent margins)(following Boeda^48^).

The presence of Neanderthal fossils, the overall character of stone tool technology present, and the faunal record indicating the presence of glacial microfauna support longstanding suggestions that the Shukbah D occupation is comparable to other Levantine Neanderthal sites and later Middle Palaeolithic sites dating to Marine Isotope Stages 4 and 3. We have studied stone tool assemblages from a sample of sites in Southwest Asia that either preserve Neanderthal fossils, are Middle Palaeolithic assemblages dating to Marine Isotope Stages 4 and 3 or both. Supplementary Table 14 provides a detailed summary of the artefacts employed within comparative studies, with key references for interpretation of the sites, fossil hominins and stone tool assemblages. For brevity, we refer to these collectively as late Middle Palaeolithic comparative assemblages. We have studied an expanded range of sites that span eastern Africa and South West Asia, including additional Middle Palaeolithic or Middle Stone Age sites dating to the Late Pleistocene, associated with *Homo sapiens* fossils, or preserve a prominent component of Nubian Levallois technology. Supplementary Table 15 provides a detailed summary of the artefacts employed within the widest comparative studies conducted here, with key references for interpretation of the sites, fossil hominins and stone tool assemblages. For brevity, we refer to these collectively as Late Pleistocene comparative assemblages.

**Methods**

We recorded a series of metric and categorical variables, which are described at length elsewhere (^49–52^). Metric variables, including maximum dimensions, axial dimensions (length; proximal, medial, distal and tip widths and thicknesses) and platform dimensions were measured using Vernier callipers, with interior and exterior platform angles measured using a goniometer. Morphological indices used here include elongation (Length/Width), Proximal Shape (Proximal/Medial Width), Distal Shape (Medial/Distal Width), Flattening (Medial Width/Thickness), Maximum Flatness (Max Dimension/Max Thickness) and Tip Cross Sectional Area (TCSA; Distal Width x Distal Thickness^53^). Choice of variables employed in multivariate analyses is guided by examination of covariance matrices with the aim of ensuring different discrete domains of technological activity are reflected. Axial Length, Elongation, Proximal Shape, Distal Shape, Flattening, TCSA, external platform angle and Platform Thickness are employed to evaluate Levallois Point attribute datasets from Shukbah and for comparisons with late Middle Palaeolithic Levantine assemblages. Maximum length, Proximal Shape, Distal Shape, Elongation, Flattening and Levallois Scar Platform Angle are employed to evaluate Levallois Point Core attribute datasets from Shukbah and for comparisons with late Middle Palaeolithic comparative assemblages. To enable comparability with the full range of Late Pleistocene comparative assemblages, Axial Length, Elongation, Proximal Shape, Maximum Flatness and Platform Thickness are used for Levallois Points. Qualitative variables recorded include blank type (Flake, Blade, Point), dorsal scar directions, flaking surface scar pattern, core typology and blank typology, including whether artefacts meet definitions for Nubian Levallois technology set out above.

All analyses were conducted using R. 4.0.2. *bestNormalize*^54^ was employed to make appropriate transformation to the quantitative data for multivariate analysis. We employed *prcomp* to conduct singular value decomposition principal components analysis on the centred and scaled datasets. *Factoextra*^55^ has been used to extract eigenvalues and explained variance data, as well as to visualise the results of the analysis for illustrations in the SI; figures presented in the main text were produced using base R packages. All Levallois Point, or all Levallois Core datasets were analysed to examine variability within the Shukbah D Assemblage. For comparative analyses with other sites, the relevant Shukbah D data were excluded from the initial analysis, and added as supplementary variables that are scaled, centred and rotated according to the results of analysis of the comparative sample using *predict*.

**Analysis of Shukbah D Assemblage**

Of the 100 cores studied here, 49 Levallois flake cores, 33 Levallois point cores (including Nubian Levallois point cores), and 1 Levallois blade core were identified. An individual non-Levallois blade core and Multi-Platform core were identified, along with a further 15 cores which show residual features suggesting discard following Levallois reduction sequences, but lacking all features required for classification as a Levallois core. Amongst the 607 debitage elements studied, Levallois points (including Nubian Levallois points) dominate, totalling 231, with a further 189 Levallois flakes, and 71 Levallois blades present. Amongst the non-Levallois debitage, 57 flakes, 49 blades and 7 points were identified.

Our analysis of the Shukbah D collections identified 12 examples of Nubian Levallois points, and 219 other Levallois point types. Nine Nubian Levallois points present bidirectional dorsal scar patterns, with the remaining three artefacts showing a combination of distal and lateral dorsal scars. Other Levallois points typically present either unidirectional convergent (n=106), bidirectional (n=49), or unidirectional (n=41) dorsal scar patterns, with more limited numbers (n=23) showing centripetal flaking. Descriptive statistics of attributes used either directly in multivariate analyses or through the production of key indices (e.g. elongation) for all Levallois points from Shukbah are presented in table Supplementary Table 16. The results of PCA for all Shukbah points is illustrated in Supplementary Figure 3 and the percentage variance and explained and variable loading results of Principal Components Analysis of all Levallois points from Shukbah D are shown in Supplementary Table 17 and Supplementary Table 18. These results indicate that Axial Length, a proxy for overall artefact size, plays a key role in driving PC1, supplemented by flattening and elongation. PC2 is predominately driven by Distal TCSA values, supplemented by Platform Thickness and elongation. Proximal shape drives PC3, whereas distal shape drives PC4, supplemented by Axial Length. Low sample sizes prohibit meaningful hypothesis testing approaches to examining variability, but it is evident that Nubian Levallois points share a common multivariate distribution that is encompassed by that of other Levallois points at Shukbah D.

Our analysis of the Shukbah D collections identified 16 Nubian Levallois point cores (Supplementary Figure 2). Three cores present only proximal and distal divergent shaping of the flaking surface, with the remaining cores showing orthagonal or centripetal shaping of the flaking surface. Sixty-eight complete Levallois cores preserve alternate flaking surface scar patterns, which do not clearly produce a medial distal ridge, including centripetal (n=47), bidirectional (n=9), unidirectional convergent (n=7), orthogonal (n=2) and unidirectional (n=1). Descriptive statistics of attributes used in analysis of Shukbah D Levallois Cores are shown in Supplementary Table 19. The results of PCA for all Shukbah Levallois cores is illustrated in Supplementary Figure 4 and the percentage variance and explained and variable loading results of Principal Components Analysis of all Levallois cores from Shukbah D are shown in Supplementary Table 20 and Supplementary Table 21. The results indicate that the combination of Proximal Shape and Elongation drive PC1, with Distal Shape and LSPA driving PC2, which combined 51% of variance within the dataset. Notably, Maximum Dimension only plays a substantive role in driving PC3, suggesting that core shape plays a more dominant role in explaining diversity than core size. Considerable similarity is observed between Nubian Levallois Point Cores and Other Levallois Point cores, which appear more distinct from with Levallois Flake cores or the sole example of a Levallois blade core. This result supports assessment of the blank dataset, suggesting that at Shukbah, Nubian Levallois reduction strategies form part of wider Levallois point production strategies, rather than a discrete technological approach.

**Comparisons between Shukbah D and late Middle Palaeolithic comparative assemblages**

No examples of Nubian Levallois points were encountered in the examination of the studied samples from late Middle Palaeolithic comparative assemblages. The majority of Levallois points preserved convergent or unidirectional dorsal scar patterns, with fewer bidirectional points, and rare centripetal points (Supplementary Table 22). The percentage variance and explained and variable loading results of Principal Components Analysis of all Levallois points from late Middle Palaeolithic Levantine sites excluding Shukbah D are shown in Supplementary Table 23 and Supplementary Table 24. The results of PCA are illustrated in Supplementary Figure 5, with Nubian Levallois and Other Levallois points from Shukbah added as supplementary variables. Axial Length, as a gross metric of size, plays a prominent but not dominant role in explaining variability across this dataset, unlike the Shukbah D assemblage, with TCSA making a more substantial contribution to PC1, which may also indicate the influence of both size and functional factors. Distal shape and elongation play prominent roles in driving PC2, which again differs from the results of analysis of the Shukbah D assemblage. It is notable that while shape and size variables play prominent roles in driving the first four principal components, platform characteristics are only notable for PC5, explaining ~13% of variability. Differentiation between all but two assemblages largely tracks differences in PC1, suggesting differences in artefact sizes, rather than shape. Levallois points from Bisitun and Tor Sabiha differ from this trend, reflecting a tendency towards higher elongation values. The distribution of the Shukbah D Levallois point data largely overlaps with that of other late Middle Palaeolithic comparative assemblages, suggesting that the impact of a selective recovery of artefacts has had rather limited impact on artefact diversity, and particularly given the notably larger sample of artefacts examined here from Shukbah. Nine of the Nubian Levallois points fall within the scope of variability observed amongst other late Middle Palaeolithic comparative Levallois points, with three artefacts marginally beyond this range, rather than forming a discrete grouping.

Examination of Levallois cores from other late Middle Palaeolithic comparative assemblages led to the identification of a stepped Nubian Levallois point core from Bisitun F. This a notable comparison to Shukbah D as another South West Asian site that has yielded a Neanderthal fossil in association with the Middle Palaeolithic stone tool assemblage. All other assemblages examined within this group lacked Nubian Levallois technology. Levallois Point cores are relatively rare in available dataset, with the majority Levallois cores having flake removals as their final Levallois scar (Supplementary Table 25). The percentage variance and explained and variable loading results of Principal Components Analysis of all Levallois point cores from late Middle Palaeolithic comparative assemblages excluding Shukbah D are shown in Supplementary Table 26 and Supplementary Table 27. The results of PCA are illustrated in Supplementary Figure 6, with Nubian Levallois and Other Levallois cores from Shukbah added as supplementary variables. Levallois Point cores from Kebara and Ksar Akil show partially overlapping distributions for PC1 and PC2. Notably, both Nubian Levallois Point cores and other Levallois point cores from Shukbah predominately overlap the distribution of Ksar Akil Levallois point cores, rather than Kebara.

The percentage variance and explained and variable loading results of Principal Components Analysis of all Levallois cores from late Middle Palaeolithic comparative assemblages excluding Shukbah D are shown in Supplementary Table 28 and Supplementary Table 29. The results of PCA are illustrated in Supplementary Figure 7, with Nubian Levallois and Other Levallois cores from Shukbah added as supplementary variables. These results complement the evaluation from the Shukbah dataset, which highlights that shape variables, rather than size, play dominant roles in explaining multivariate variability. Levallois flake cores exhibit greater variability in PC1, whereas differences in distal shape drives differences between flake and points cores in PC2. The Nubian Levallois point core from Bisitun falls within the wider variability of Levallois Point cores observed. Core data from Shukbah largely overlap with variability observed across late Middle Palaeolithic assemblages examined here, with Levallois Point cores forming a subset of variability within that observed from other sites. All but one Nubian Levallois Point core overlap with variability observed amongst other Levallois Point Cores across late Middle Palaeolithic assemblages studied. This result supports the evaluation of blank datasets to suggest that both Nubian Levallois Point and Point Cores fall within variability observed across other Levallois Point reduction approaches identified from multiple late Middle Palaeolithic assemblages.

**Comparisons between Shukbah D and other Late Pleistocene comparative assemblages**

Seventeen Nubian Levallois points are present in the dataset analysed from TH123b, with a further twelve Nubian Levallois points present at TH383, with each site yielding two examples of other Levallois point types. No Nubian Levallois points were identified amongst the Qafzeh samples examined, where points typically occur at low frequency in contrast to flakes and exclusively preserve unidirectional or convergent dorsal scar patterns. The percentage variance and explained and variable loading results of Principal Components Analysis of all Levallois points from Late Pleistocene comparative assemblages excluding Shukbah D are shown in Supplementary Table 30 and Supplementary Table 31. The results of PCA are illustrated in Supplementary Figure 8 and Supplementary Figure 9 and indicate that differences between Levallois points from the southern Arabian sites of TH123b and TH383 are distinct from other Levallois points in the Late Pleistocene comparative sample. This difference can largely be explained by gross differences in artefact sizes, potentially highlighting the use of the Nubian reduction method in the context of immediate raw material availability and with limited impacts from reduction intensity. PCA results with Shukbah dated added as a supplementary variable are illustrated in Supplementary Figure 8. Levallois points from Shukbah overlap with the range of variability observed for PC1 and PC2 amongst the comparative dataset with the exception of nine artefacts, with a further three observed beyond the range of variability amongst Other Levallois points but within the variability observed amongst Nubian Levallois points. The Nubian Levallois points from Shukbah all overlap with variability observed in Other Levallois points, with half of the Nubian Levallois points from Shukbah also overlap the range of variability of Levallois points observed at TH123b and TH383. We interpret this result to suggest that although some Nubian Levallois points from Shukbah may result from early phases of reduction sequences, comparable to those observed in southern Arabian sites, the Nubian Levallois points from Shukbah may illustrate the use of this reduction strategy in later phases of reduction sequences too, leading to greater comparability to the wider Levallois point datasets.

Nubian Levallois Point cores dominant the examined southern Arabian assemblages from TH123b and TH383 (Supplementary Table 32), whereas the majority of additional Late Pleistocene comparative datasets examined here predominately or exclusively comprise Levallois Flake cores. The percentage variance explained and variable loading results of Principal Components Analysis of all Levallois points from Late Pleistocene comparative assemblages excluding Shukbah D are shown in Supplementary Table 33 and Supplementary Table 34. The results of PCA are illustrated in Supplementary Figure 10. These results support the importance of shape variables over size variables in explaining variability, though notably in contrast to examination of the late Middle Palaeolithic dataset, the wider sample included here results in a more prominent role for Maximum Dimension in PC2. In a scenario comparable to the blank assemblage, Levallois cores from TH123b and TH383, which are almost exclusively Nubian Levallois Point cores, show a distinct distribution in contrast to the remainder of the comparative dataset, reflecting both larger ranges and average values for the Distal Shape index, Elongation and Maximum Dimension. While it is evident that key elements of core shaping drive this distinct distribution, the extent to which size differences may indicate the differential impact of reduction intensity enabling different core morphologies warrants future exploration. The results of PCA are illustrated in Supplementary Figure 11, with Nubian Levallois and Other Levallois cores from Shukbah added as supplementary variables. These results further demonstrate that variability in the Shukbah assemblage falls within the range observed amongst other Late Pleistocene sites comparative assemblages, suggesting that selective artefact curation may have had more limited impact on artefact variability than might otherwise be assumed. Levallois Flake and Point cores from Shukbah exhibit a subset of variability evident across the comparative sample. Nine of the fourteen Nubian Levallois point cores from Shukbah share a common range in PC1 and PC 2 values to other Nubian Levallois point cores examined, whereas all but one fall within the range of values observed amongst Other Levallois Point cores. We interpret the results of the foregoing analysis to suggest that Nubian Levallois technology is not a discrete from the wider range of variability observed in Levallois Point production methods.

**Supplementary Table 1 ǀ** **Discrete morphological tooth crown non-metric traits assessed in EM 3869 and compared with Pleistocene and Holocene hominins.**

| **Trait** | **Shukbah** | | **Grade** | | **Neanderthals** | | **Early *Homo sapiens*** | | **Western Asia Upper Palaeolithic modern humans** | **European Upper Palaeolithic modern humans** | **Natufians** | **Holocene humans** | |
| --- | --- | --- | --- | --- | --- | --- | --- | --- | --- | --- | --- | --- | --- |
|  | **OES** | **EDJ** | **OES** | **EDJ** | **OES N (%)** | **EDJ N (%)** | **OES N (%)** | **EDJ N (%)** | **OES N (%)** | **OES N (%)** | **OES N (%)** | **OES N (%)** | **EDJ N (%)** |
| anterior fovea | 4 | large | >1 | large | 31/35 (89%)^f^ |  | 11/15 (73%)^b1,b2^ |  | 0/4 (0%)^c^ | 10/19 (53%)^f^ |  | 2/114 (2%)^a^ |  |
| mid-trigonid crest | 1B | 2 | >0 | >1 | 37/39 (95%)^e^ | 29/30 (99%)^d^ | 5/16 (31%)^e^ | 0/5 (0%)^b2^ | 0/4 (0%)^c^ | 0/24 (0%)^f^ | 0/119 (0%)^g^ | 210/3402 (6%)^h^ | 8/29 (28%)^b2,d^ |
| groove pattern | X |  | non-Y |  | 1/37 (3%)^f^ |  | 1/9 (11%)^b1^ |  | 0/5 (0%)^c^ | 2/28 (7%)^f^ |  | 24/87 (28%)^a^ |  |
| cusp 5 | 4 | 4 | >0 | >3 | 48/49 (98%)^f^ | 16/18 (89%)^i-k^ | 13/13 (100%)^b1^ | 3/3 (100%)^i-k^ | 5/5 (100%)^c^ | 34/35 (97%)^f^ |  | 4498/4548 (99%)^h^ | 29/37 (78%)^i-k^ |
| cusp 6 | – | 0 | =0 | =0 | 17/25 (68%)^e^ | 12/18 (67%)^i-k^ | 14/16 (88%)^e^ | 3/3 (100%)^i-k^ | 3/4 (75%)^c^ | 18/22 (82%)^f^ | 126/156 (81%)^g^ | 2556/4548 (56%)^h^ | 29/37 (78%)^i-k^ |
| cusp 7 | 1 | 1 | >0 | >0 | 12/40 (30%)^e^ | 3/18 (17%)^i-k^ | 11/23 (48%)^e^ | 2/3 (67%)^i-k^ | 1/4 (25%)^c^ | 2/29 (7%)^f^ | 5/170 (3%)^g^ | 567/6191 (9%)^h^ | 3/36 (8%)^i-k^ |

^a 56^; ^b1^Supplementary Table 5; ^b2^Supplementary Table 8; ^c^ Supplementary Table 6; ^d 34^; ^e 57^; ^f 58^; ^g 59^(cusp 7, grades >1); ^h 31^; ^I 60^; ^j 61^; ^k^A. Ortiz, pers. comm.;

**Supplementary Table 2 ǀ Dimensions of the M_1_ EM 3869 (in mm).**

| **dimension** | **value** |
| --- | --- |
| Crown ‒ mesiodistal, actual | 13.5 |
| Crown ‒ mesiodistal, corrected | 13.7 |
| Crown ‒ buccolingual | 12.0 |
| Crown area | 164 |
| Crown index | 88 |
| Crown height (maximum) | 7.3 |
| Cervix ‒ mesiodistal | 11.2 |
| Cervix ‒ buccolingual | 11.0 |
| Cervix ‒ root robusticity | 123 |
| Stem length ‒ buccal | 5.8 |
| Root length | 13.5 |

**Supplementary Table 3 ǀ Comparative crown dimensions (in mm).**

| **sample** |  | ***n*** | **MD length** | **BL breadth** |
| --- | --- | --- | --- | --- |
| EM 3869 |  |  | 13.7 | 12.0 |
| Neanderthals^a^ | mean | 34 | 11.8 | 11.1 |
|  | SD |  | 0.9 | 0.7 |
|  | range |  | 10.1‒13.6 | 9.7‒12.9 |
|  | Azs |  | **1.0** | 0.6 |
| Western Asian Neanderthals^b^ | mean | 9 | 11.2 | 10.8 |
|  | SD |  | 0.6 | 0.5 |
|  | range |  | 10.5‒12.3 | 10.0‒11.5 |
|  | Azs |  | **1.8** | **1.0** |
| early *H. sapiens*^c^ | mean | 27 | 12.5 | 11.6 |
|  | SD |  | 1.0 | 0.7 |
|  | range |  | 10.5‒14.5 | 10.0‒12.9 |
|  | Azs |  | 0.6 | 0.3 |
| Western Asia Upper Palaeolithic modern humans^d^ | mean | 10 | 11.7 | 11.0 |
|  | SD |  | 0.7 | 0.6 |
|  | range |  | 10.9−13.1 | 10.1−12.1 |
|  | Azs |  | **1.3** | 0.7 |
| Natufian^e^ | mean | 101 | 11.36 | 11.01 |
|  | SD |  | 0.66 | 0.52 |
|  | Azs |  | **1.8** | 0.9 |

Abbreviations: Azs = Adjusted Z score; BL = buccolingual; MD = mesiodistal; X = sample mean; SD = sample standard deviation; *n* = sample size.

^a^ Data source: ^57^. ^b^ Data source: Supplementary Table 4. ^c^ Data source: Supplementary Table 5. ^d^ Data source: Supplementary Table 6. ^e^ Data source: ^62^

**Supplementary Table 4 ǀ Western Asian Neanderthal metric samples and sources.**

| **site** | **specimen numbers** | **source** |
| --- | --- | --- |
| Amud | 1 | ^63^ |
| Dederiyeh | 1 | ^64^ |
| Kebara | 2 | ^65^ |
| Shanidar | 1, 2 | ^66^ |
| Tabun | T1 | ^67^ |
|  | Layer B series 3, Layer Eb | ^8^ |
| Teshik-Tash | 1 | ^67^ |

**Supplementary Table 5 ǀ Early *H. sapiens* samples and sources.**

| **site** | **specimen numbers** | **source (metrics & morphology)** |
| --- | --- | --- |
| Dar es-Soltan 2 | H4, H5 | ^68^ |
| Jebel Irhoud | 3, 11 | ^57^ |
| Smuggler's Cave (Témara) | mandible, 7a | ^68^ |
| El Haroura (Zouhrah) | mandible | ^68^ |
| Skhūl | 1, 4, 5, 6, 7, 10 | ^8^ |
| Qafzeh | 4, 7, 8, 9, 10, 11, 12, 15, 16, 21 | ^69–71^ ^(metrics only)^ |
| Die Kelders Cave | AP6242, AP6277 | ^72,73^ |
| Equus Cave | H5 | ^74^ |
| Klasies River Mouth | 13400, 14696 | ^75^ |

**Supplementary Table 6 ǀ Western Asia Upper Palaeolithic modern human samples and sources.**

| **site** | **specimen numbers** | **source (metrics)** | **source (morphology)** |
| --- | --- | --- | --- |
| El Wad Aurignacian | D1, E mandible, E loose | Original specimens measured | Original specimens studied |
| Kebara Aurignacian | D3, D4, D5 | Original specimens measured | Original specimens studied |
| Ksar Akil | 1 | ^76^  ^77^  ^78^  ^79^ |  |
| Neve David |  |  |  |
| Qasr Kharaneh | IV (H2) |  |  |
| Wadi Mataha | F-81 |  |  |

**Supplementary Table 7** **|** **Tooth crown tissue proportions of EM 3869 compared with fossil and extant hominin M1 samples and adjusted Z-score statistics (Azs) of the Shukbah specimen compared with the two hominin groups**. 3D LAET: three-dimensional lateral average enamel thickness (mm); 3D LRET: three-dimensional lateral relative enamel thickness. The Azs results suggest that the 3D LAET and 3D LRET values of EM 3869 are statistically compatible with the range of variation of both comparative groups.

|  |  | **3D LAET** | **3D LRET** | **Azs(3D LAET)** | **Azs(3D LRET)** |
| --- | --- | --- | --- | --- | --- |
| EM 3869 |  | 0.59 | 9.03 |  |  |
| Neanderthals (n=10) | mean | 0.58 | 8.67 | 0.04 | 0.12 |
|  | s.d. | 0.11 | 1.30 |  |  |
| Holocene humans (n=13) | mean | 0.56 | 11.81 | 0.15 | -0.36 |
|  | s.d. | 0.09 | 3.42 |  |  |

**Supplementary Table 8 | Pleistocene and Holocene hominin comparative microtomographic data used for the 3D geometric morphometric analysis (3D GMA) of the enamel-dentine junction (EDJ).**

| **specimen** | **locality (country)** | **group** | **n** | **references** |
| --- | --- | --- | --- | --- |
| S5 | La Chaise-de-Vouthon Abri Suard (France) | Neanderthal | 1 | ^80^ |
| S49 | La Chaise-de-Vouthon Abri Suard (France) | Neanderthal | 1 | ^80^ |
| S14-7 | La Chaise-de-Vouthon Abri Suard (France) | Neanderthal | 1 | ^<sup>80,81</sup><sup>80,81</sup>80,81^ |
| Archi 1 | Archi (Italy) | Neanderthal | 1 | ^original data^ |
| BD-J4-C9 | La Chaise-de-Vouthon Abri Bourgeois-Delaunay (France) | Neanderthal | 1 | ^80,81^ |
| KRD77 | Krapina (Croatia) | Neanderthal | 1 | ^80^ |
| KRD79 | Krapina (Croatia) | Neanderthal | 1 | ^80^ |
| KRD81 | Krapina (Croatia) | Neanderthal | 1 | ^80^ |
| KRD105 | Krapina (Croatia) | Neanderthal | 1 | ^80^ |
| Ehringsdorf I | Ehringsdorf (Germany) | Neanderthal | 1 | ^80^ |
| Fossellone 3 | Fossellone (Italy) | Neanderthal | 1 | ^original data^ |
| Gibraltar 2 | Devil's Tower (Gibraltar) | Neanderthal | 1 | ^82^ |
| Molare 1 | Molare (Italy) | Neanderthal | 1 | ^original data^ |
| Lagar Velho 1 | Lagar Velho (Portugal) | Pleistocene modern human | 1 | ^83^ |
| Qafzeh 4 | Qafzeh (Israel) | Pleistocene modern human | 2 | ^original data^ |
| Qafzeh 10 | Qafzeh (Israel) | Pleistocene modern human | 1 | ^82^ |
| Qafzeh15 | Qafzeh (Israel) | Pleistocene modern human | 2 | ^<sup>82</sup><sup>82</sup>82^ |
| Holocene humans | Eurasia | Holocene humans | 14 | ^80^ |

**Supplementary Table 9 | Results of the cross-validated between-group principal component analyses (bgPCA).** The leave-one-out cross-validation (CV) shows excellent predictive accuracy, with a clear discrimination between Neanderthals and modern humans, even if Pleistocene modern humans and Holocene humans tend to overlap. The posterior probabilities that EM 3869 is closer to any of the comparative groups were then calculated. The highest probability is indicated in bold (the greater the number, the higher the probability).

|  | **correct classification after CV** | **posterior probability for EM 3869** |
| --- | --- | --- |
| Neanderthals | 92.3% | **0.433** |
| Pleistocene modern humans | 66.7% | <0.001 |
| Holocene humans | 100.0% | <0.001 |

**Supplementary Table 10 | Results of the canonical variates analyses (CVA).** The Jackknife cross-validation (CV) shows good predictive accuracy with a clear discrimination between Neanderthals, Pleistocene modern humans and Holocene humans. The posterior probabilities that EM 3869 belongs to any of the comparative groups were then calculated.

|  | **correct classification after CV** | **posterior probability for EM 3869** |
| --- | --- | --- |
| Neanderthals | 92.3% | **100.0%** |
| Pleistocene modern humans | 100.0% | 0.0% |
| Holocene humans | 100.0% | 0.0% |

**Supplementary Table 11 ǀ Comparative root lengths (in mm)**.^a^

| **sample** |  | ***n*** | **root length** |
| --- | --- | --- | --- |
| EM 3869 |  |  | 13.2 |
| Neanderthals | mean | 19 | 15.6 |
|  | SD |  | 1.6 |
|  | Azs |  | -0.7 |
| early *H. sapiens* | mean | 11 | 15.4 |
|  | SD |  | 1.8 |
|  | Azs |  | -0.5 |
| recent *H. sapiens* | mean | 12 | 13.7 |
|  | SD |  | 0.9 |
|  | Azs |  | -0.2 |

Abbreviations: Azs = Adjusted Z score; X = sample mean; SD = sample standard deviation; *n* = sample size.

^a^ Data source: ^44^

**Supplementary Table 12** **|** **Volumetric bifurcation index (VBI) of EM 3869 compared with fossil and extant hominin M1 samples and adjusted Z-score statistics (Azs) of the Shukbah specimen compared with the two hominin groups**. The values highlighted in bold (above 1.00) indicate that the VBI value of EM 3869 statistically exceeds the 95% confidence interval of the comparative groups.

|  |  | **VBI** | **Azs(VBI)** |
| --- | --- | --- | --- |
| EM 3869 |  | 68.20 |  |
| Neanderthals (n=19) | mean | 54.62 | 0.68 |
|  | s.d. | 9.33 |  |
| Pleistocene modern humans (n=11) | mean | 48.29 | **1.31** |
|  | s.d. | 6.51 |  |
| Holocene humans (n=12) | mean | 49.27 | **1.14** |
|  | s.d. | 7.25 |  |

**Supplementary Table 13:** **Distribution of Shukbah D artefacts across global institutions as identified by Callander**^5^

| Total selected and reported by Garrod | 1235 |
| --- | --- |
| Institute of Archaeology, UCL | 493 |
| British Museum | 72 |
| Institut de Paléontologie Humaine, Paris | 34 |
| Museum of Archaeology and Anthropology, University of Cambridge | 61 |
| Pitt Rivers Museum, University of Oxford | 73 |
| University Museum, University of Manchester | 70 |
| Rockefeller Museum, Jerusalem | 126 |
| Peabody Museum, Harvard University | 103 |
| Redpath Museum, McGill University | 54 |
| Royal Ontario Museum | 72 |
| Untraced | 77 |
| Total Traced (Callander) | 1158 |

**Supplementary Table 14: Details of assemblages employed in comparisons between Shukbah D and late Middle Palaeolithic comparative assemblages.**

| Site | Assemblage | Fossils | Chronology | All Levallois Points | All Levallois Cores |
| --- | --- | --- | --- | --- | --- |
| Bisitun^84,85^ | F | Neanderthal^85^ |  | 21 | 9 |
| Kebara^86^ | X | Neanderthal | 61.1 ± 4 ka^87^; 60 ± 6ka/ 64 ± 6 ka^88^ | 48 | 11 |
|  | XI | Neanderthal | 60 ± 3.5 ka^87^ | 39 | 14 |
|  | XII | Neanderthal | 59.5 ± 3.5 ka^87^ | 10 | 2 |
| Ksar Akil^89,90^ | XXVI |  | 47 ± 9 ka^91^; <44.3-42.5 ka^92^ | 36 | 24 |
|  | XXVIII |  | <44.3-42.5 ka^92^ (minimum age) | - | 54 |
| Rosh Ein Mor^93^ |  |  | ~70-34ka^94^ | 27 | 8 |
| Tor Faraj^95^ | Floor 2 |  | 42.-69ka^95,96^ | 45 | 8 |
| Tor Sabiha^97^ | C |  | 69ka^96^ | 16 | 4 |

**Supplementary Table 15: Details of additional assemblage samples employed in comparisons between Shukbah D and Late Pleistocene comparative assemblages**.

| Site | Assemblage | Fossils | Date | All Levallois Points | All Levallois Cores |
| --- | --- | --- | --- | --- | --- |
| TH123b^15^ |  |  |  | 19 | 52 |
| Aduma^98^ | A5 |  | 80-100 ka |  | 78 |
| Al Marrat^99^ | ALM3 |  |  |  | 19 |
| Al Wusta^100^ |  | *Homo Sapiens* | ~86-95 ka |  | 54 |
| Omo^101^ | BNS |  | ~104 ± 1 ka |  | 24 |
| JKF1^102^ |  |  | 49 ± 5 to 87± 6 ka |  | 29 |
| Mundafan^103^ | MDF61 |  |  |  | 88 |
| Qafzeh ^104^ | XVII | *Homo Sapiens* | 92 ± 5ka | 2 | 7 |
| Qafzeh ^104^ | XIX | *Homo Sapiens* | 92 ± 5ka | 10 | 10 |
| Skhul^105^ |  | *Homo Sapiens* | 102 ± 26ka to 119 ± 18ka |  | 48 |
| TH383^106^ |  |  |  | 14 | 53 |

**Supplementary Table 16**: Descriptive statistics of attributes used in analysis of Shukbah D Levallois points, differentiating Nubian and Other Levallois points.

|  | Variable | N | Min | Median | Max | Mean | SD | SE |
| --- | --- | --- | --- | --- | --- | --- | --- | --- |
| Nubian Levallois | Axial Length | 12 | 48.73 | 65.41 | 86.16 | 65.44 | 10.69 | 3.09 |
|  | Prox Axial Width |  | 18.94 | 31.01 | 41.79 | 30.94 | 6.77 | 1.95 |
|  | Med Axial Width |  | 21.27 | 28.145 | 37.07 | 28.53 | 4.94 | 1.43 |
|  | Dist Axial Width |  | 12.32 | 16.42 | 25.98 | 17.24 | 4.63 | 1.34 |
|  | Med Thickness |  | 3.06 | 6.33 | 8.08 | 5.98 | 1.61 | 0.47 |
|  | Dist Thickness |  | 3.21 | 5.615 | 9.36 | 5.73 | 1.95 | 0.56 |
|  | Platform Thickness |  | 3.27 | 4.885 | 8.2 | 5.07 | 1.43 | 0.41 |
|  | EPA |  | 64 | 72 | 81 | 72.17 | 5.37 | 1.55 |
| Other Levallois | Axial Length | 219 | 33.84 | 56.42 | 99.39 | 57.17 | 11.10 | 0.75 |
|  | Prox Axial Width |  | 14.91 | 33.81 | 58.52 | 34.22 | 7.51 | 0.51 |
|  | Med Axial Width |  | 11.4 | 29.4 | 47.72 | 29.91 | 6.61 | 0.45 |
|  | Dist Axial Width |  | 8.35 | 17.64 | 34.58 | 17.88 | 4.50 | 0.30 |
|  | Med Thickness |  | 3.2 | 6.36 | 15.85 | 6.58 | 1.90 | 0.13 |
|  | Dist Thickness |  | 1.58 | 5.01 | 11.76 | 5.36 | 1.67 | 0.11 |
|  | Platform Thickness |  | 2.28 | 5.6 | 12.74 | 5.82 | 1.89 | 0.13 |
|  | EPA |  | 44 | 75 | 170 | 75.11 | 10.84 | 0.73 |

**Supplementary Table 17**: Eigenvalues and percentage of variance explained by each Principal Component from analysis of the Shukbah D Levallois Point dataset.

|  | Eigenvalue | % Variance Explained | Cumulative % Variance Explained |
| --- | --- | --- | --- |
| PC1 | 1.711665 | 21.39582 | 21.39582 |
| PC2 | 1.555 | 19.4375 | 40.83331 |
| PC3 | 1.338166 | 16.72707 | 57.56038 |
| PC4 | 1.293435 | 16.16793 | 73.72832 |
| PC5 | 0.932777 | 11.65971 | 85.38803 |
| PC6 | 0.679633 | 8.495407 | 93.88344 |
| PC7 | 0.402185 | 5.027314 | 98.91075 |
| PC8 | 0.08714 | 1.08925 | 100 |

**Supplementary Table 18**: Principal component loadings from analysis of the Shukbah D Levallois Point dataset, with the top two contributing variables highlighted in bold for principal components with eigen values greater than 1.

|  | PC1 | PC2 | PC3 | PC4 | PC5 | PC6 | PC7 | PC8 |
| --- | --- | --- | --- | --- | --- | --- | --- | --- |
| Axial Length | **0.54** | -0.02 | 0.28 | **0.46** | 0.02 | 0.36 | 0.06 | -0.53 |
| Elongation | 0.45 | -0.53 | 0.27 | 0.06 | -0.11 | -0.25 | 0.29 | 0.53 |
| Prox Shp | 0.23 | -0.06 | **-0.66** | -0.03 | 0.36 | 0.49 | 0.27 | 0.25 |
| Dist Shp | -0.24 | -0.16 | -0.08 | **0.73** | 0.27 | -0.05 | -0.47 | 0.29 |
| Flattening | **-0.51** | 0.03 | **0.42** | 0.14 | -0.12 | 0.55 | 0.42 | 0.23 |
| Distal TCSA | 0.37 | **0.60** | 0.23 | -0.12 | -0.08 | 0.23 | -0.38 | 0.49 |
| EPA | -0.01 | 0.09 | 0.39 | -0.19 | 0.87 | -0.15 | 0.12 | -0.03 |
| Platform Thickness | 0.03 | **0.56** | -0.16 | 0.42 | -0.04 | -0.43 | 0.54 | 0.07 |

**Supplementary Table 19**: Descriptive statistics for Attributes/Indices used to compare Nubian Levallois and Other Levallois cores from Shukbah D.

|  |  | n | Min | Median | Max | Mean | SD | SE |
| --- | --- | --- | --- | --- | --- | --- | --- | --- |
| Nubian Levallois | Max Dimension | 16 | 45.74 | 51.145 | 64.27 | 53.08 | 5.49 | 1.37 |
|  | Axial Length | 16 | 40.66 | 48.925 | 58.24 | 49.05 | 4.86 | 1.21 |
|  | Prox Axial Width | 16 | 28.57 | 40.05 | 55.16 | 40.58 | 7.42 | 1.86 |
|  | Med Axial Width | 16 | 32.04 | 42.685 | 61.49 | 42.62 | 7.08 | 1.77 |
|  | Dist Axial Width | 16 | 18.62 | 29.315 | 37.88 | 29.65 | 6.05 | 1.51 |
|  | Med Thickness | 16 | 13.19 | 18.7 | 35.08 | 20.81 | 6.32 | 1.58 |
|  | IPA | 16 | 54 | 65 | 86 | 67.88 | 10.15 | 2.54 |
| Other Levallois | Max Dimension | 66 | 35.73 | 54.54 | 81.95 | 56.46 | 9.78 | 1.20 |
|  | Axial Length | 66 | 33.86 | 47.93 | 73.96 | 49.73 | 9.08 | 1.12 |
|  | Prox Axial Width | 66 | 27.59 | 41.655 | 65.41 | 43.29 | 8.65 | 1.06 |
|  | Med Axial Width | 66 | 31.25 | 46.37 | 69.9 | 49.22 | 10.11 | 1.24 |
|  | Dist Axial Width | 66 | 19.03 | 37.575 | 63.07 | 38.06 | 9.60 | 1.18 |
|  | Med Thickness | 66 | 9.91 | 20.435 | 44.49 | 21.70 | 7.71 | 0.95 |
|  | IPA | 66 | 35 | 68 | 109 | 69.79 | 12.28 | 1.51 |

**Supplementary Table 20**: Eigenvalues and percentage of variance explained by each Principal Component from analysis of the Shukbah D Levallois Point dataset.

|  | Eigenvalue | % Variance Explained | Cumulative % Variance Explained |
| --- | --- | --- | --- |
| PC1 | 1.887379 | 31.45632 | 31.45632 |
| PC2 | 1.233427 | 20.55712 | 52.01344 |
| PC3 | 1.017003 | 16.95005 | 68.96349 |
| PC4 | 0.795048 | 13.2508 | 82.21429 |
| PC5 | 0.580528 | 9.675475 | 91.88977 |
| PC6 | 0.486614 | 8.110232 | 100 |

**Supplementary Table 21**: Principal component loadings from analysis of the late Middle Palaeolithic Levantine Levallois Point dataset, with the top two contributing variables highlighted in bold for principal components with eigen values greater than 1.

|  | PC1 | PC2 | PC3 | PC4 | PC5 | PC6 |
| --- | --- | --- | --- | --- | --- | --- |
| Max Length | 0.28 | 0.18 | **-0.77** | 0.45 | -0.05 | 0.30 |
| Prox Shp | **-0.54** | 0.13 | -0.19 | -0.45 | -0.39 | 0.55 |
| Dist Shp | -0.30 | **0.64** | -0.30 | -0.21 | 0.36 | -0.48 |
| Elongation | **-0.52** | -0.26 | 0.03 | 0.34 | 0.67 | 0.31 |
| Flattening | 0.48 | 0.37 | 0.25 | -0.33 | 0.44 | 0.51 |
| LSPA | 0.17 | **-0.58** | **-0.47** | -0.57 | 0.28 | -0.13 |

**Supplementary Table 22**: Dorsal scar patterns of Levallois points from late Middle Palaeolithic Levantine sites employed in comparative analyses.

|  | Bidirectional | Centripetal | Convergent | Unidirectional |
| --- | --- | --- | --- | --- |
| Bisitun_F | 2 | 0 | 4 | 15 |
| Kebara_X | 3 | 4 | 37 | 4 |
| Kebara_XI | 6 | 0 | 22 | 11 |
| Kebara_XII | 3 | 0 | 3 | 4 |
| Ksar Akil_XXVI | 2 | 7 | 20 | 7 |
| Rosh Ein Mor | 1 | 0 | 20 | 6 |
| Tor Faraj_Floor2 | 5 | 2 | 29 | 9 |
| Tor Sabiha_C | 4 | 1 | 5 | 6 |

**Supplementary Table 23**: Eigenvalues and percentage of variance explained by each Principal Component from analysis of the late Middle Palaeolithic Levantine Levallois Point dataset.

|  | Eigenvalue | % Variance Explained | Cumulative % Variance Explained |
| --- | --- | --- | --- |
| PC1 | 1.949551 | 24.36938 | 24.36938 |
| PC2 | 1.437853 | 17.97316 | 42.34254 |
| PC3 | 1.250547 | 15.63183 | 57.97438 |
| PC4 | 1.094737 | 13.68422 | 71.65859 |
| PC5 | 1.032468 | 12.90585 | 84.56444 |
| PC6 | 0.765428 | 9.567846 | 94.13229 |
| PC7 | 0.411364 | 5.142053 | 99.27434 |
| PC8 | 0.058053 | 0.725657 | 100 |

**Supplementary Table 24**: Principal component loadings from analysis of the late Middle Palaeolithic Levantine Levallois Point dataset, with the top two contributing variables highlighted in bold for principal components with eigen values greater than 1.

|  | PC1 | PC2 | PC3 | PC4 | PC5 | PC6 | PC7 | PC8 |
| --- | --- | --- | --- | --- | --- | --- | --- | --- |
| Axial Length | **0.52** | -0.33 | 0.05 | -0.28 | 0.00 | 0.51 | 0.14 | 0.51 |
| Elongation | 0.09 | **-0.72** | 0.20 | 0.36 | 0.04 | 0.16 | -0.23 | -0.48 |
| ProxShp | -0.21 | 0.06 | **-0.65** | -0.23 | 0.31 | 0.52 | -0.20 | -0.27 |
| DistShp | -0.26 | **-0.40** | -0.15 | **-0.54** | -0.42 | -0.18 | 0.45 | -0.22 |
| Flattening | -0.14 | 0.23 | **0.60** | **-0.50** | -0.15 | 0.28 | -0.41 | -0.21 |
| DistalTCSA | **0.62** | 0.34 | 0.03 | -0.05 | 0.07 | 0.03 | 0.39 | -0.58 |
| EPA | 0.14 | -0.22 | 0.06 | -0.42 | **0.69** | -0.50 | -0.16 | 0.01 |
| Platform Thickness | 0.44 | 0.00 | -0.37 | -0.11 | **-0.47** | -0.29 | -0.59 | -0.01 |

**Supplementary Table 25:** Late Middle Palaeolithic Levallois core blank types from sampled assemblages.

|  | Blade | Flake | Point | Nubian |
| --- | --- | --- | --- | --- |
| Bisitun_F | 0 | 8 | 0 | 1 |
| Kebara_X | 0 | 5 | 6 | 0 |
| Kebara_XI | 0 | 11 | 3 | 0 |
| Kebara_XII | 0 | 1 | 1 | 0 |
| Ksar Akil_XXVI | 0 | 23 | 1 | 0 |
| Ksar Akil XXVIII | 0 | 25 | 29 | 0 |
| Rosh Ein Mor | 0 | 7 | 1 | 0 |
| Tor Faraj_Floor2 | 1 | 6 | 1 | 0 |
| Tor Sabiha_C | 0 | 4 | 0 | 0 |

**Supplementary Table 26:** Eigenvalues and percentage of variance explained by each Principal Component from analysis of the late Middle Palaeolithic Levallois Point Core comparative dataset.

|  | Eigenvalue | % Variance Explained | Cumulative % Variance Explained |
| --- | --- | --- | --- |
| PC1 | 1.759316 | 29.32193 | 29.32193 |
| PC2 | 1.231804 | 20.53007 | 49.852 |
| PC3 | 1.179296 | 19.65493 | 69.50693 |
| PC4 | 0.887699 | 14.79498 | 84.30191 |
| PC5 | 0.607491 | 10.12485 | 94.42677 |
| PC6 | 0.334394 | 5.573231 | 100 |

**Supplementary Table 27:** Principal component loadings from analysis of the late Middle Palaeolithic Levallois Point Core comparative dataset, with the top two contributing variables highlighted in bold for principal components with eigen values greater than 1.

|  | PC1 | PC2 | PC3 | PC4 | PC5 | PC6 |
| --- | --- | --- | --- | --- | --- | --- |
| Max Length | -0.03 | **-0.55** | **-0.53** | 0.39 | 0.51 | -0.01 |
| Prox Shp | -0.38 | 0.30 | -0.15 | 0.75 | -0.42 | 0.07 |
| Dist Shp | **-0.49** | -0.52 | 0.24 | -0.10 | -0.29 | -0.58 |
| Elongation | **-0.57** | -0.12 | -0.32 | -0.44 | -0.13 | 0.59 |
| Flattening | 0.43 | **-0.56** | 0.22 | 0.18 | -0.47 | 0.44 |
| IPA | 0.33 | 0.05 | **-0.70** | -0.23 | -0.48 | -0.33 |

**Supplementary Table 28:** Eigenvalues and percentage of variance explained by each Principal Component from analysis of the late Middle Palaeolithic Levallois Core comparative dataset.

|  | Eigenvalue | % Variance Explained | Cumulative % Variance Explained |
| --- | --- | --- | --- |
| PC1 | 1.597931 | 26.63218 | 26.63218 |
| PC2 | 1.308972 | 21.8162 | 48.44838 |
| PC3 | 1.022254 | 17.03757 | 65.48595 |
| PC4 | 0.827048 | 13.78413 | 79.27008 |
| PC5 | 0.688742 | 11.47904 | 90.74912 |
| PC6 | 0.555053 | 9.250879 | 100 |

**Supplementary Table 29**: Principal component loadings from analysis of the late Middle Palaeolithic Levallois Core comparative dataset, with the top two contributing variables highlighted in bold for principal components with eigen values greater than 1.

|  | PC1 | PC2 | PC3 | PC4 | PC5 | PC6 |
| --- | --- | --- | --- | --- | --- | --- |
| Max Length | 0.01 | 0.18 | **-0.92** | 0.25 | -0.24 | -0.03 |
| Prox Shp | 0.46 | 0.27 | -0.22 | -0.70 | 0.36 | -0.23 |
| Dist Shp | 0.04 | **0.71** | 0.11 | 0.36 | 0.44 | 0.40 |
| Elongation | **0.58** | 0.07 | **0.19** | 0.51 | -0.08 | -0.60 |
| Flattening | **-0.63** | 0.07 | -0.07 | 0.08 | 0.46 | -0.62 |
| LSPA | 0.24 | **-0.62** | -0.23 | 0.25 | 0.63 | 0.22 |

**Supplementary Table 30**: Eigenvalues and percentage of variance explained by each Principal Component from analysis of the Late Pleistocene Middle Palaeolithic Levallois Point dataset.

|  | Eigenvalue | % Variance Explained | Cumulative % Variance Explained |
| --- | --- | --- | --- |
| PC1 | 1.934086 | 38.68172 | 38.68172 |
| PC2 | 1.100843 | 22.01687 | 60.69859 |
| PC3 | 0.94369 | 18.8738 | 79.57239 |
| PC4 | 0.825645 | 16.51291 | 96.0853 |
| PC5 | 0.195735 | 3.9147 | 100 |

**Supplementary Table 31**: Principal component loadings from analysis of the Late Pleistocene Middle Palaeolithic Levallois Point dataset, with the top two contributing variables highlighted in bold for principal components with eigen values greater than 1.

|  | PC1 | PC2 | PC3 | PC4 | PC5 |
| --- | --- | --- | --- | --- | --- |
| Axial Length | **-0.53** | -0.46 | -0.37 | -0.17 | 0.58 |
| Elongation | -0.48 | 0.41 | -0.30 | -0.55 | -0.46 |
| Prox Shp | 0.31 | **-0.47** | 0.36 | -0.74 | -0.08 |
| Max Flat | 0.39 | -0.39 | -0.73 | 0.10 | -0.40 |
| Platform Thickness | **-0.50** | **-0.50** | 0.34 | 0.33 | -0.53 |

**Supplementary Table 32:** Late Pleistocene Middle Palaeolithic Levallois core blank types from sampled assemblages.

|  | Blade | Flake | Point | Nubian |
| --- | --- | --- | --- | --- |
| TH123b | 0 | 0 | 1 | 51 |
| A5 | 1 | 68 | 7 | 0 |
| ALM3 | 0 | 19 | 0 | 0 |
| AlWusta | 0 | 54 | 0 | 0 |
| Bisitun_F | 0 | 8 | 0 | 1 |
| BNS | 0 | 22 | 0 | 0 |
| JKF1 | 0 | 29 | 0 | 0 |
| Kebara_X | 0 | 5 | 6 | 0 |
| Kebara_XI | 0 | 11 | 3 | 0 |
| Kebara_XII | 0 | 1 | 1 | 0 |
| Ksar Akil_XXVI | 0 | 23 | 1 | 0 |
| KsarAkil28A | 0 | 25 | 29 | 0 |
| MDF61 | 0 | 88 | 0 | 0 |
| Qafzeh_XIX | 0 | 10 | 0 | 0 |
| Qafzeh_XVII | 0 | 7 | 0 | 0 |
| Rosh Ein Mor | 0 | 7 | 1 | 0 |
| Skhul | 0 | 40 | 4 | 0 |
| TH383 | 1 | 8 | 5 | 38 |
| Tor Faraj_Floor2 | 1 | 6 | 1 | 0 |
| Tor Sabiha_C | 0 | 4 | 0 | 0 |

**Supplementary Table 33**: Eigenvalues and percentage of variance explained by each Principal Component from analysis of the Late Pleistocene Middle Palaeolithic Levallois Point dataset.

|  | Eigenvalue | % Variance Explained | Cumulative % Variance Explained |
| --- | --- | --- | --- |
| PC1 | 1.807421 | 36.14841 | 36.14841 |
| PC2 | 1.222184 | 24.44368 | 60.59209 |
| PC3 | 0.925402 | 18.50804 | 79.10014 |
| PC4 | 0.590203 | 11.80405 | 90.90419 |
| PC5 | 0.45479 | 9.095809 | 100 |

**Supplementary Table 34**: Principal component loadings from analysis of the late Middle Palaeolithic Levantine Levallois Point dataset, with the top two contributing variables highlighted in bold for principal components with eigen values greater than 1.

|  | PC1 | PC2 | PC3 | PC4 | PC5 |
| --- | --- | --- | --- | --- | --- |
| Max Length | -0.44 | **0.58** | -0.05 | -0.42 | -0.54 |
| Prox Shp | -0.33 | -0.36 | 0.76 | -0.41 | 0.13 |
| Dist Shp | **-0.54** | 0.21 | 0.27 | 0.77 | 0.05 |
| Elongation | **-0.55** | -0.07 | -0.5 | -0.24 | 0.62 |
| Flattening | 0.31 | **0.69** | 0.31 | -0.12 | 0.56 |

**
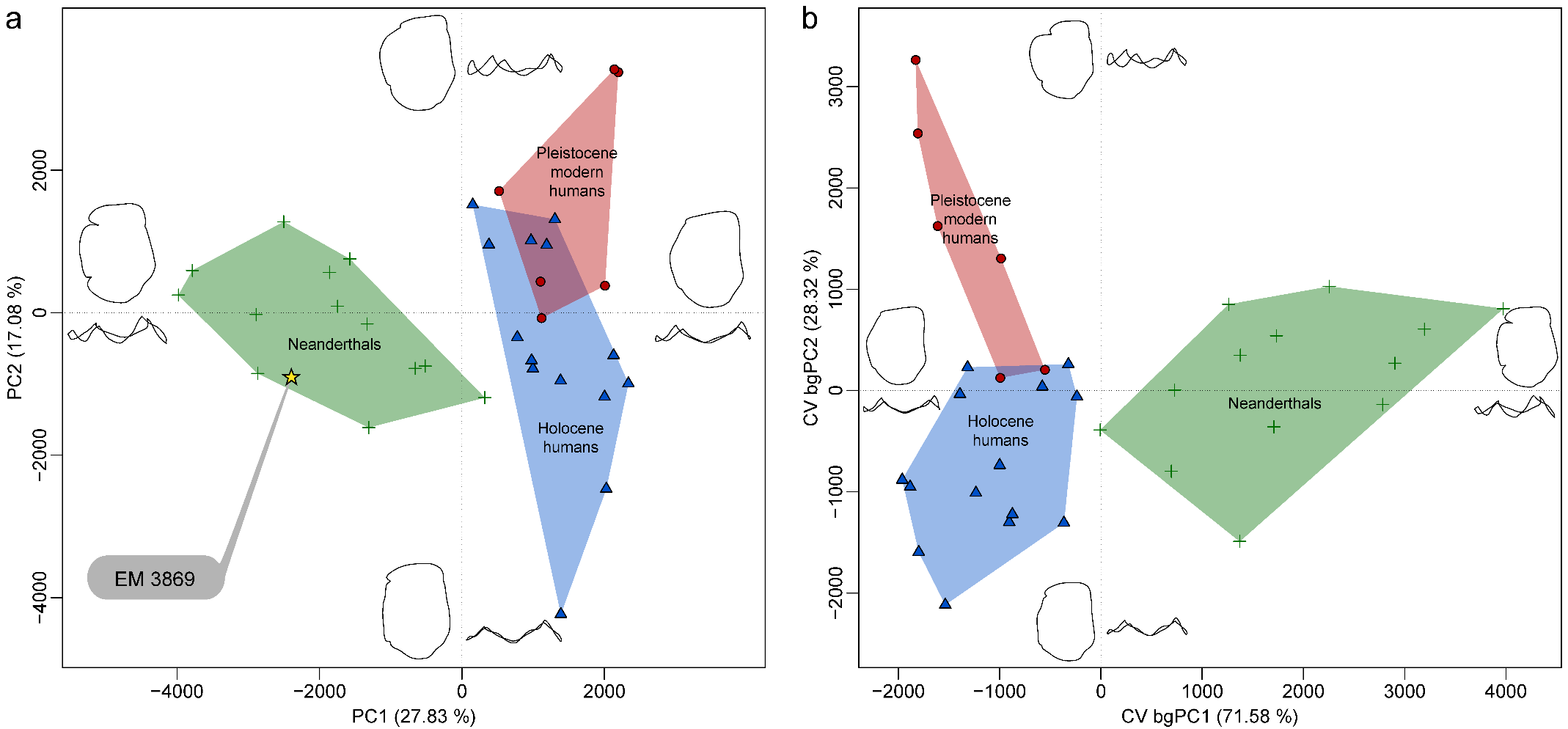
**

**Supplementary Figure 1 | Geometric morphometric analyses of the enamel-dentine junction (EDJ) shape of EM 3869. a,** Principal components analyses based on the 3D semilandmarks Procrustes-registered shape coordinates of the EDJ of EM 3869 compared with fossil and extant hominins (SI Table 8). **b,** Cross-validated bgPCA based on the 3D semilandmarks Procrustes-registered shape coordinates of the EDJ of the comparative fossil and extant hominin groups showing a similar distribution to that in the normal bgPCA (Figure 3b). The wireframes at the end of the axes illustrate the extreme morphological variation trends in occlusal (mesial aspect upward and buccal on the right) and buccal views (mesial aspect rightward).


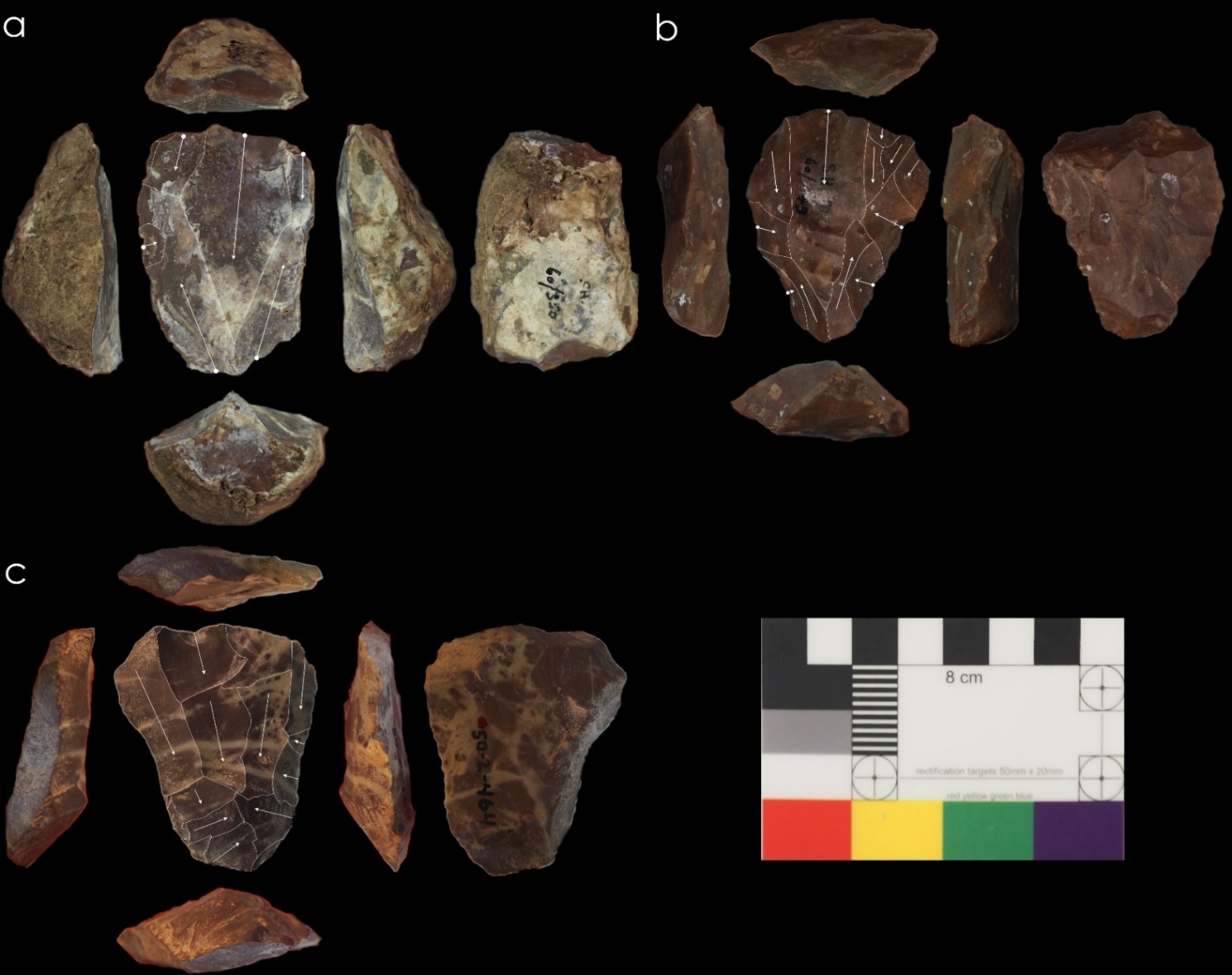


**Supplementary Figure 2 |** Nubian Levallois point cores from Shukbah (a-b; © UCL, Institute of Archaeology) and Bisitun (c; courtesy of the Penn Museum, University of Pennsylvania).


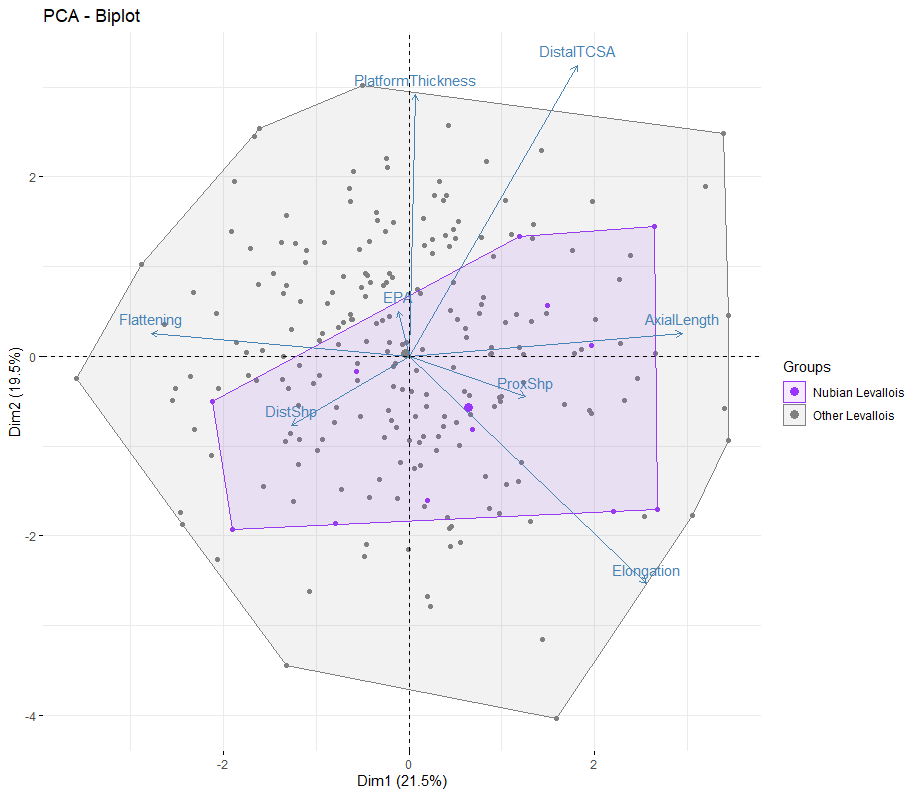


**Supplementary Figure 3 |** Biplot of first two principal components of analysis of all Levallois points from Shukbah D, highlighting differences between Nubian and other Levallois points


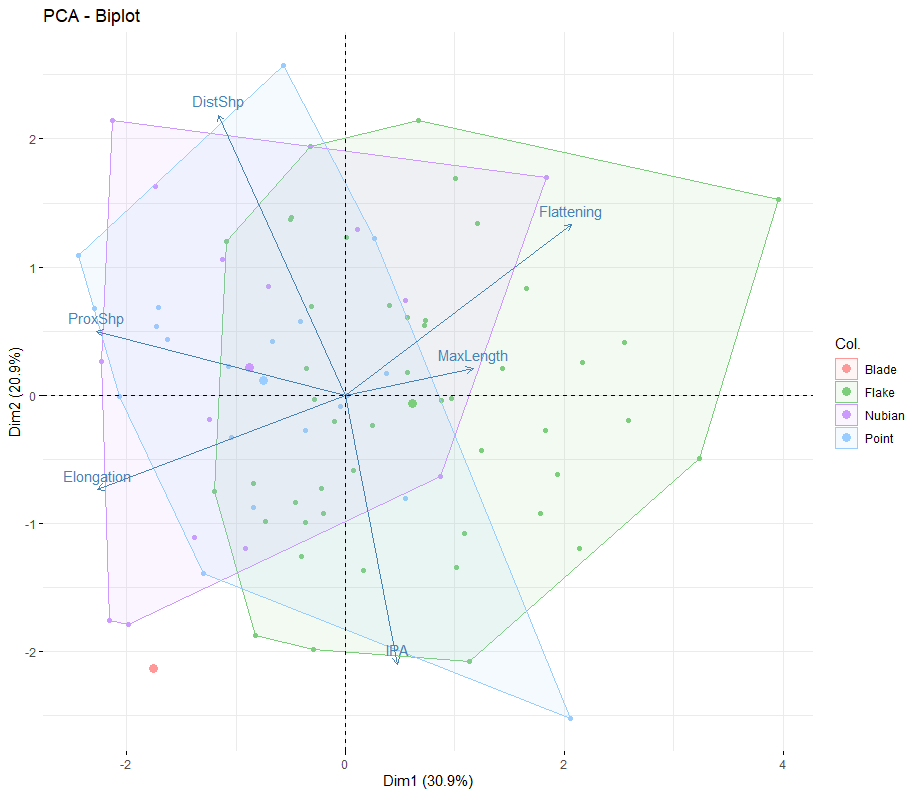


**Supplementary Figure 4 |** Biplot of first two principal components of analysis of all Levallois Cores from Shukbah D, highlighting differences between Nubian and other Levallois Point, Flake and Blade cores.


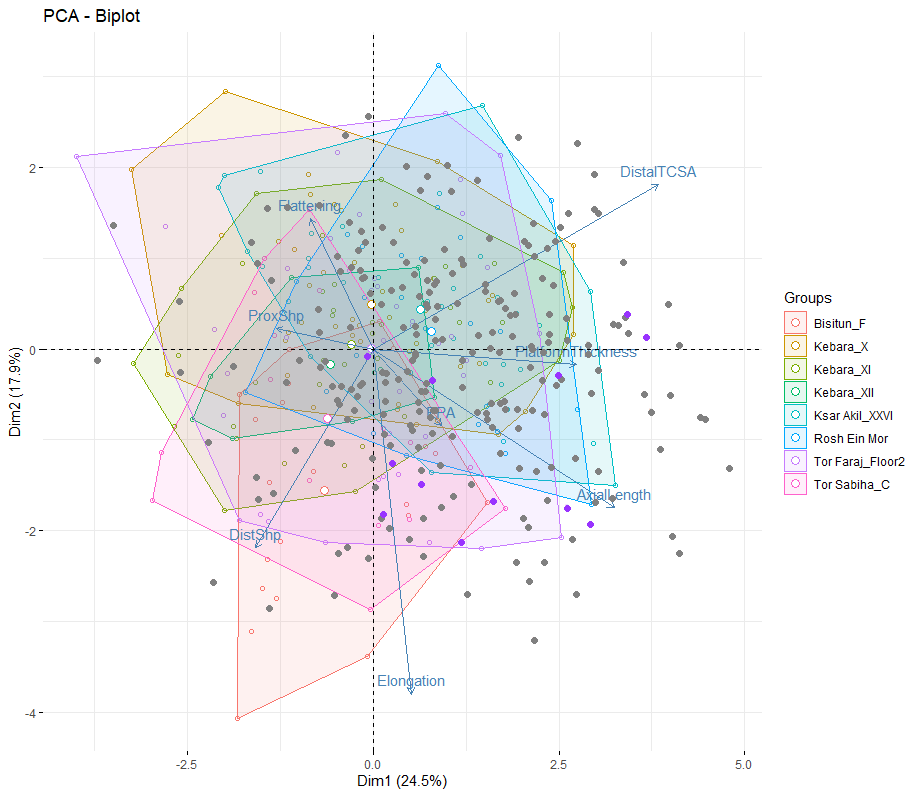


**Supplementary Figure 5 |** Biplot of first two principal components of analysis of all Levallois points from late Middle Palaeolithic comparative sites, with Shukbah D artefacts added as a supplementary variable; Shukbah D Nubian Levallois points shown in solid purple, and Other Levallois points shown in solid grey.


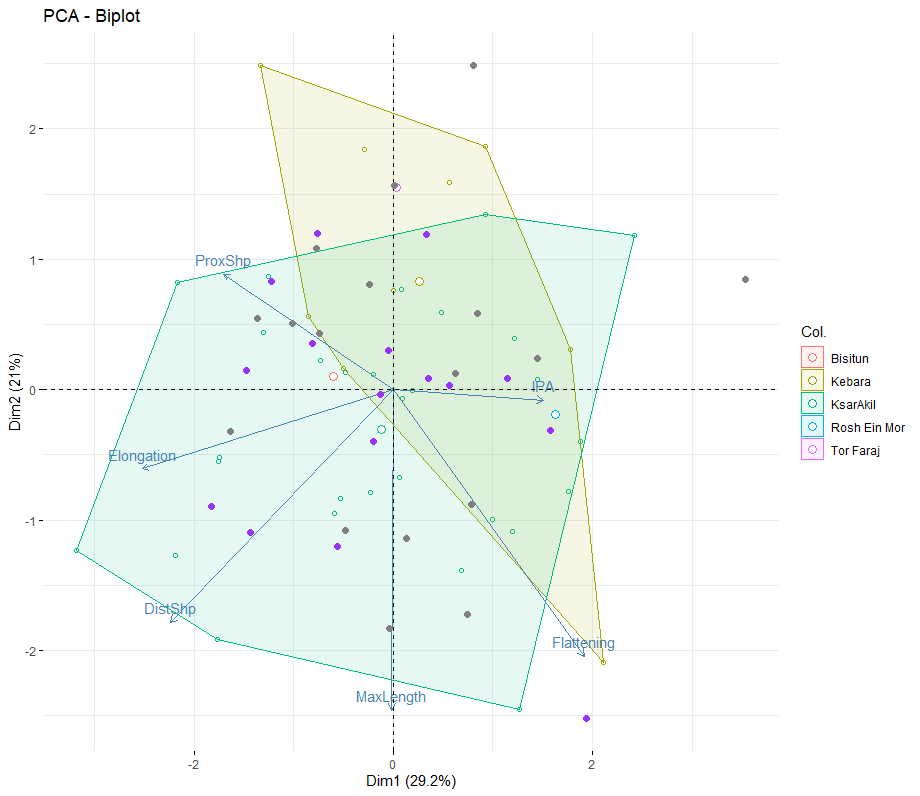


**Supplementary Figure 6 |** Biplot of first two principal components of analysis of all Levallois Point Cores from late Middle Palaeolithic comparative sites, with Shukbah D artefacts added as a supplementary variable; Shukbah D Nubian Levallois points shown in solid purple, and Other Levallois points shown in solid grey.


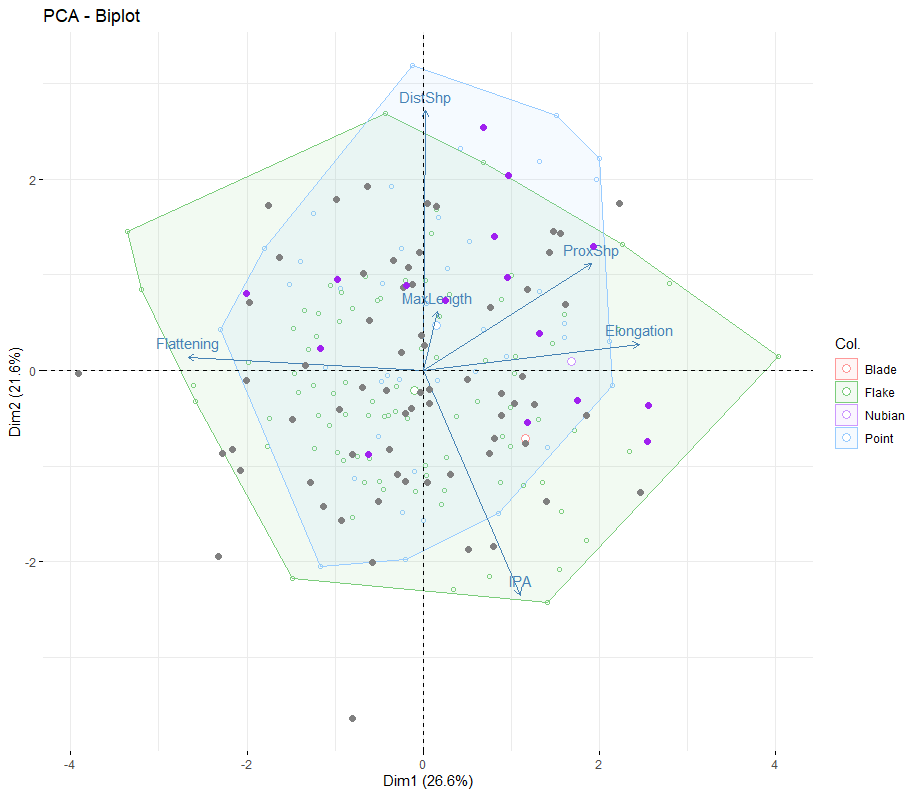


**Supplementary Figure 7 |** Biplot of first two principal components of analysis of all Levallois Cores from late Middle Palaeolithic comparative sites, with Shukbah D artefacts added as a supplementary variable; Shukbah D Nubian Levallois points shown in solid purple, and Other Levallois points shown in solid grey.


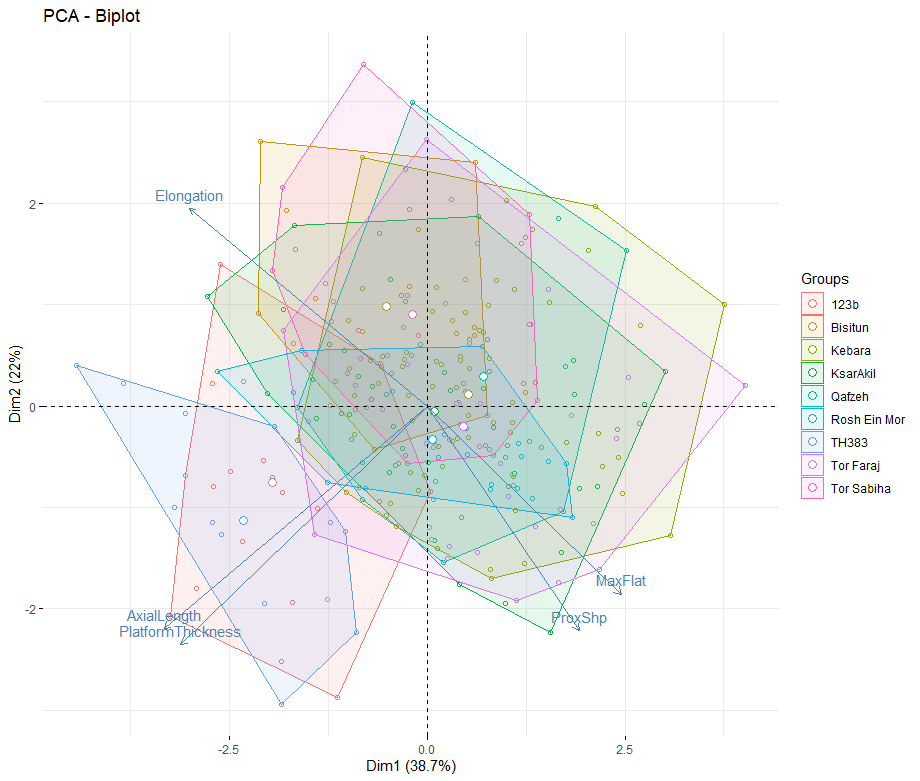


**Supplementary Figure 8 |** Biplot of first two principal components of analysis of all Levallois points from Late Pleistocene comparative sites.


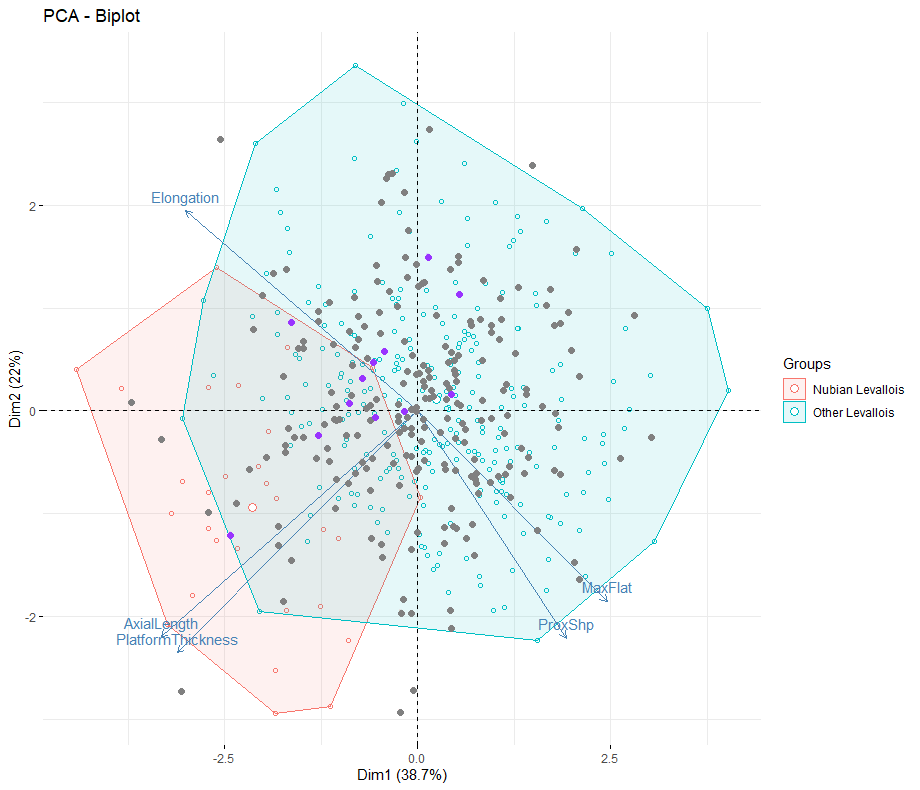


**Supplementary Figure 9 |** Biplot of first two principal components of analysis of all Levallois Points from Late Pleistocene comparative sites, with Shukbah D artefacts added as a supplementary variable; Shukbah D Nubian Levallois points shown in green, and Other Levallois points shown in grey.


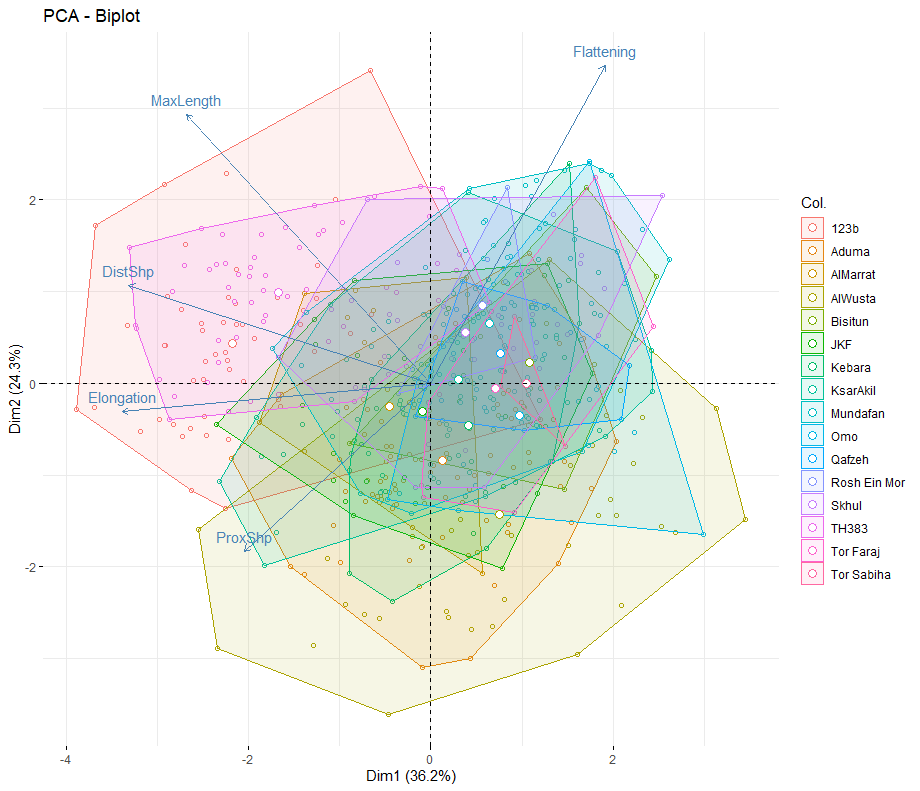


**Supplementary Figure 10 |** Biplot of first two principal components of analysis of all Levallois cores from Late Pleistocene comparative sites.


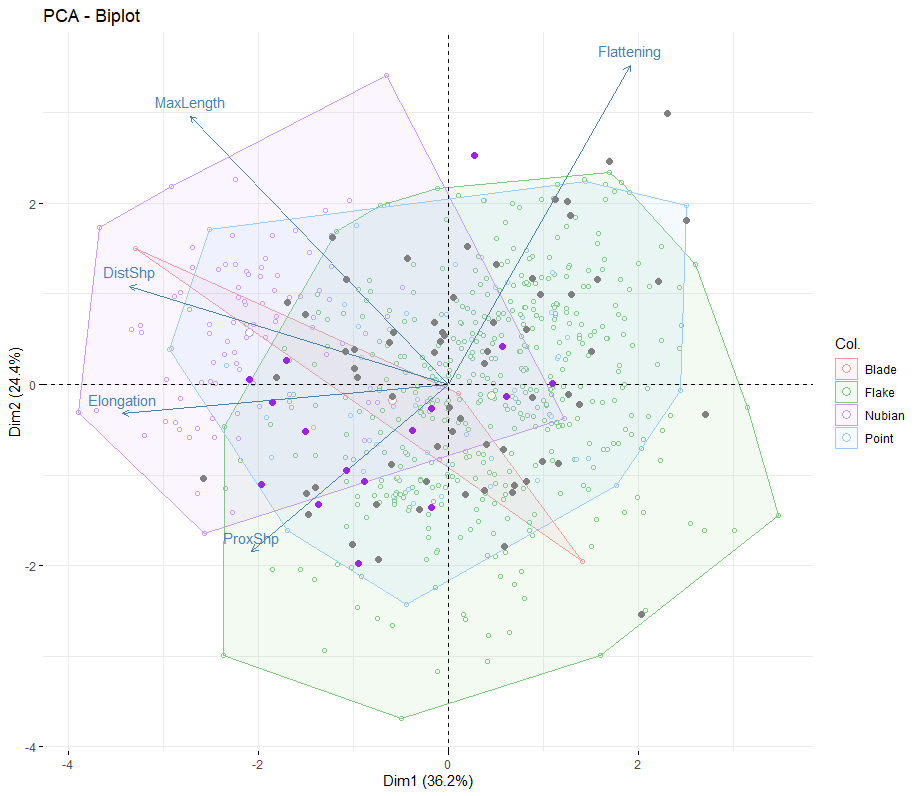


**Supplementary Figure 11 |** Biplot of first two principal components of analysis of all Levallois Cores from Late Pleistocene comparative sites, with Shukbah D artefacts added as a supplementary variable; Shukbah D Nubian Levallois Point cores shown in solid purple, Other Levallois types are shown in solid grey.

**References Cited :**

1. Mallon, A. Quelques stations prehistorique de Palestine. *Melanges de I’Universite Saint-Joseph* **10**, 191 (1924).

2. Frumkin, A., Langford, B., Marder, O. & Ullman, M. Paleolithic caves and hillslope processes in south-western Samaria, Israel: Environmental and archaeological implications. *Quat. Int.* **398**, 246–258 (2016).

3. Garrod, D. A. E. Excavation of a Palaeolithic Cave in Western Judaea. *Palest. Explor. Q.* **60**, 182–185 (1928).

4. Garrod, D. A. E. & Bate, D. M. A. Excavations at the Cave of Shukbah, Palestine, 1928. *Proc. Prehist. Soc.* **8**, 1–20 (1942).

5. Callander, J. Dorothy Garrod ’ s Excavations in the Late Mousterian of Shukbah Cave in Palestine Reconsidered. *Proc. Prehist. Soc.* **70**, 207–231 (2004).

6. Garrod, D. A. E. Shukba 1928. in *Unpublished handwritten excavation diary, 4 April - 2 June. In: Box 63 [Inventory no. 33 431], Garrod Archive in the Fonds Suzanne Cassou de St. Mathurin, Musée des Antiquitiés Nationales, St. Germain-en-Laye 78103, France.*

7. Keith, A. *New Discoveries relating to the antiquity of man*. (WW Norton Incorporated, 1931).

8. McCown, T. D. & Keith, A. *The Stone Age of Mount Carmel Vol. 2: the Fossil Human Remains from the Levalloiso-Mousterian*. (1939).

9. De Groote, I., Bello, S. M., Kruszynski, R., Compton, T. & Stringer, C. Sir Arthur Keith’s Legacy: Re-discovering a lost collection of human fossils. *Quat. Int.* **337**, 237–253 (2014).

10. Groucutt, H. S. Culture and Convergence: The Curious Case of the Nubian Complex. in *Culture History and Convergent Evolution: Can we detect populations in prehistory?* (ed. Groucutt, H. S.) 55–86 (Springer, 2020). doi:10.1007/978-3-030-46126-3_4

11. Seligman, C. G. The Older Palaeolithic Age in Egypt. *J. R. Anthropol. Inst. Gt. Britain Irel.* **51**, 115–153 (1921).

12. Guichard, J. & Guichard, G. The Early and Middle Palaeolithic of Nubia: a preliminary report. in *Contributions to the prehistory of Nubia* 57–116 (ort Burgwin res. center, Southern Methodist University, 1965).

13. Van Peer, P. The Nile Corridor and the Out‐of‐Africa Model An Examination of the Archaeological Record. *Curr. Anthropol.* **39**, S115–S140 (1998).

14. Rose, J. I. *et al.* The Nubian Complex of Dhofar , Oman : An African Middle Stone Age Industry in Southern Arabia. **6**, (2011).

15. Usik, V. I., Rose, J. I., Hilbert, Y. H., Van Peer, P. & Marks, A. E. Nubian Complex reduction strategies in Dhofar, southern Oman. *Quat. Int.* **300**, 244–266 (2013).

16. Crassard, R. & Hilbert, Y. H. A Nubian complex site from central Arabia: implications for Levallois taxonomy and human dispersals during the upper Pleistocene. *PLoS One* **8**, e69221 (2013).

17. Vermeersch, P. M., Paulissen, E., Stokes, S., Charlier, C. & Lindsay, W. A Middle Palaeolithic burial of a modern human at Taramsa Hill , Egypt. **72**, 475–484 (1998).

18. Will, M., Mackay, A. & Phillips, N. Implications of Nubian-like core reduction systems in southern Africa for the identification of early modern human dispersals. *PLoS One* **10**, 1–21 (2015).

19. Hallinan, E. & Shaw, M. *Nubian Levallois reduction strategies in the Tankwa Karoo, South Africa*. *PLoS ONE* **15**, (2020).

20. Blinkhorn, J., Achyuthan, H., Petraglia, M. & Ditchfield, P. Middle palaeolithic occupation in the thar desert during the upper pleistocene: The signature of a modern human exit out of Africa? *Quat. Sci. Rev.* **77**, (2013).

21. Blinkhorn, J., Achyuthan, H. & Ajithprasad, P. Middle Palaeolithic point technologies in the Thar Desert, India. *Quat. Int.* **382**, (2015).

22. Bilsborough, A. & Thompson, J. L. The dentition of the Le Moustier 1 Neanderthal. in *The neanderthal adolescent Le Moustier 1 : new aspects, new results* 157–186 (Staatliche Museen Zu Berlin - Preussicher Kulturbesitz, 2005).

23. Hillson, S. & Bond, S. Relationship of enamel hypoplasia to the pattern of tooth crown growth: A discussion. *Am. J. Phys. Anthropol.* **104**, 89–103 (1998).

24. AlQahtani, S. J., Hector, M. P. & Liversidge, H. M. Brief communication: The London atlas of human tooth development and eruption. *Am. J. Phys. Anthropol.* **142**, 481–490 (2010).

25. Smith, T. M., Toussaint, M., Reid, D. J., Olejniczak, A. J. & Hublin, J. J. Rapid dental development in a Middle Paleolithic Belgian Neanderthal. *Proc. Natl. Acad. Sci. U. S. A.* **104**, 20220–20225 (2007).

26. Smith, T. M. *et al.* Dental evidence for ontogenetic differences between modern humans and Neanderthals. *Proc. Natl. Acad. Sci. U. S. A.* **107**, 20923–20928 (2010).

27. Shaw, J. C. M. Taurodont Teeth in South African Races. *J. Anat.* **62**, 476–498 (1928).

28. Keene, H. A morphometric and biometric study of taurodontism in a contemporary population. *Am. J. Phys. Anthropol.* **25**, 208–209 (1966).

29. Rosas, A. *et al.* The growth pattern of Neandertals, reconstructed from a juvenile skeleton from El Sidrón (Spain). *Science (80-. ).* **359**, 1282–1287 (2017).

30. Scott, G. R. & Turner, C. G. *Anthropology of modern human teeth*. (Cambridge University Press, 1997).

31. Scott, G. R. & Irish, J. D. *Human Tooth Crown and Root Morphology*. (Cambridge University Press, 2017).

32. Zubov, A. A. The epicristid or middle trigonid crest defined. *Dent. Anthropol.* **6**, 9 (1992).

33. Wu, L. & Turner, C. G. Brief communication: Variation in the frequency and form of the lower permanent molar middle trigonid crest. *Am. J. Phys. Anthropol.* **91**, 245–248 (1993).

34. Bailey, S. E., Skinner, M. M. & Hublin, J. J. What lies beneath? An evaluation of lower molar trigonid crest patterns based on both dentine and enamel expression. *Am. J. Phys. Anthropol.* **145**, 505–518 (2011).

35. Martínez de Pinillos, M. *et al.* Trigonid crests expression in Atapuerca-Sima de los Huesos lower molars: Internal and external morphological expression and evolutionary inferences. *Comptes Rendus - Palevol* **13**, 205–221 (2014).

36. Kanazawa, E., Seikikawa, M., Natori, M., Kamiakito, Y. & Ozaki, T. Frequencies of Occurrence of Accessory Tubercles and Other Traits in the Lower Deciduous Second Molar. *J. Anthr. Soc. Nippon* **100**, 43–51 (1992).

37. Grine, F. E. Occlusal Morphology of the Mandibular Permanent Molars of the South African Negro and the Kalahari San (Bushman). *Ann. South African Museum* **86**, 157–215 (1981).

38. Weidenreich, F. The dentition of Sinanthoropus pekinensis. A comparative odontography of the hominids. *Paleont. Sin.* **101**, 1–180 (1937).

39. Compton, T. & Stringer, C. The human remains. in *Neanderthals in Wales: Pontnewydd and the Elwy Valley Caves* (eds. Aldhouse Green, S., Peterson, R. & Walker, E. A.) 118–230 (Oxbow Books, 2012).

40. Compton, T. & Stringer, C. The morphological affinities of the Middle Pleistocene hominin teeth from Pontnewydd Cave, Wales. *J. Quat. Sci.* **30**, 713–730 (2015).

41. Kallay, J. A new classification of the taurodont teeth of the Krapina Neanderthal Man. *Bull. Sci. Yugosl.* 2–3 (1970).

42. Shifman, A. & Chanannel, I. Prevalence of taurodontism found in radiographic dental examination of 1,200 young adult Israeli patients. *Community Dent. Oral Epidemiol.* **6**, 200–203 (1978).

43. Kupczik, K. & Hublin, J. J. Mandibular molar root morphology in Neanderthals and Late Pleistocene and recent Homo sapiens. *J. Hum. Evol.* **59**, 525–541 (2010).

44. Kupczik, K., Delezene, L. K. & Skinner, M. M. Mandibular molar root and pulp cavity morphology in Homo naledi and other Plio-Pleistocene hominins. *J. Hum. Evol.* **130**, 83–95 (2019).

45. Garrod, D. A. E. & Bate, D. M. A. *The Stone Age of Mount Carmel I*. (AMS Press, 1937).

46. Shea, J. J. *Stone Tools in the Paleolithic and Neolithic Near East*. (Cambridge University Press, 2013). doi:10.1017/CBO9781139026314

47. Copeland, L. The Middle and Upper Palaeolithic of Lebanon and Syria in lifht of recent research. in *Problems in Prehistory: North Africa and the Levant* (eds. Wendorf, F. & Marks, A. E.) 317–350 (Southern Methodist University Press, 1975).

48. Boeda, E. *Le concept Levallois, Variabilité des méthodes.* (CNRS, 1994).

49. Blinkhorn, J. Buddha Pushkar revisited: Technological variability in Late Palaeolithic stone tools at the Thar Desert margin, India. *J. Archaeol. Sci. Reports* **20**, (2018).

50. Scerri, E. M. L., Gravina, B., Blinkhorn, J. & Delagnes, A. Can Lithic Attribute Analyses Identify Discrete Reduction Trajectories? A Quantitative Study Using Refitted Lithic Sets. *J. Archaeol. Method Theory* **23**, 669–691 (2015).

51. Scerri, E. M. L., Drake, N. a., Jennings, R. & Groucutt, H. S. Earliest evidence for the structure of Homo sapiens populations in Africa. *Quat. Sci. Rev.* **101**, 207–216 (2014).

52. Scerri, E. M. L., Groucutt, H. S., Jennings, R. P. & Petraglia, M. D. Unexpected technological heterogeneity in northern Arabia indicates complex Late Pleistocene demography at the gateway to Asia. *J. Hum. Evol.* **75**, 125–142 (2014).

53. Shea, J. J. The origins of lithic projectile point technology: evidence from Africa, the Levant, and Europe. *J. Archaeol. Sci.* **33**, 823–846 (2006).

54. Peterson, R. A. Package ‘bestNormalize’. 1–27 (2020).

55. Kassambara, A. Practical guide to principal component methods in R: PCA, M (CA), FAMD, MFA, HCPC, factoextra. Vol. 2. *Sthda* (2016).

56. Martinón-Torres, M., Bermúdez De Castro, J. M., Gómez-Robles, A., Prado-Simón, L. & Arsuaga, J. L. Morphological description and comparison of the dental remains from Atapuerca-Sima de los Huesos site (Spain). *J. Hum. Evol.* **62**, 7–58 (2012).

57. Hublin, J. *et al.* New fossils from Jebel Irhoud, Morocco and the pan-African origin of Homo sapiens. *Nat. Publ. Gr.* **546**, 289–292 (2017).

58. Bailey, S. Beyond shovel-shaped incisors: Neandertal dental morphology in a comparative context. *Period. Biol.* **108**, 253–267 (2006).

59. Irish, J. D. The Iberomaurusian enigma: North African progenitor or dead end? *J. Hum. Evol.* **39**, 393–410 (2000).

60. Ortiz, A., Bailey, S. E., Hublin, J. J. & Skinner, M. M. Homology, homoplasy and cusp variability at the enamel–dentine junction of hominoid molars. *J. Anat.* **231**, 585–599 (2017).

61. Ortiz, A., Bailey, S. E., Schwartz, G. T., Hublin, J. J. & Skinner, M. M. Evo-devo models of tooth development and the origin of hominoid molar diversity. *Sci. Adv.* **4**, 1–7 (2018).

62. Smith, P. & Verdéne, J. The dentition of the PPNA specimens. in *Le gisment de Hatoula en Judée Occidentale, Isra* (eds. Lechevallier, M. & Ronen, A.) 73–79 (C.N.R.S., 1994).

63. Sakura, H. Dentition of the Amud man. in *The Amud Man and his cave site* (eds. Suzuki, H. & Takai, F.) 207–230 (University of Tokyo, 1970).

64. *Neanderthal burials. Excavations of the Dederiyeh Cave, Afrin, Syria. International Research Center for Japanese Studies, Kyoto.* (International Research Center for Japanese Studies, 2002).

65. Tillier, A., Arensburg, B., Vandermeersch, B. & Chech, M. New human remains from Kebara Cave (Mount Carmel). The place of the Kebara hominids in the Levantine Mousterian fossil record. *Paléorient* **29**, 35–62 (2003).

66. Trinkaus, E. Dental remains from the Shanidar adult Neanderthals. *J. Hum. Evol.* **7**, 369–382 (1978).

67. Frayer, D. W. *The evolution of the dentition in Upper Paleolithic and Mesolithic Europe*. (University of Kansas, 1978).

68. Hublin, J.-J. *et al.* Dental Evidence from the Aterian Human Populations of Morocco. in *Modern Origins: A North African Perspective* (eds. Hublin, J.-J. & McPherron, S. P.) 35–47 (Springer, 2012). doi:10.1007/978-94-007-2929-2

69. Vermeersch, P. M. *Les Hommes Fossiles de Qafzeh (Israel)*. (C.N.R.S., 1981).

70. Tillier, A. New Middle Palaeolithic Hominin Dental Remains from Qafzeh, Israel. *Paléorient* **40**, 13–24 (2014).

71. Tillier, A. M. *Les enfants moustériens de Qafzeh. Interprétataions phylogénétique et paléoauxologique*. (CNRS Éditions (Cahiers de Paléoanthropologie), 1999).

72. Grine, F. E., Klein, R. G. & Volman, T. P. Dating, archaeology and human fossils from the Middle Stone Age levels of Die Kelders, South Africa. *J. Hum. Evol.* **21**, 363–395 (1991).

73. Grine, F. E. Middle Stone Age human fossils from Die Kelders Cave 1, Western Cape Province, South Africa. *J. Hum. Evol.* **38**, 129–145 (2000).

74. Grine, F. E. & Klein, R. G. Pleistocene and Holocene Human Remains from Equus Cave, South Africa. *Anthropology* **8**, 55–98 (1985).

75. Rightmire, G. P. & Deacon, H. J. Comparative studies of Late Pleistocene human remains from Klasies River Mouth, South Africa. *J. Hum. Evol.* **20**, 131–156 (1991).

76. Bergman, C. A. & Stringer, C. B. Fifty years after: Egbert, an early Upper Palaeolithic juvenile from Ksar Akil, Lebanon. *Paléorient* **15**, 99–111 (1989).

77. Bocquentin, F., Crevecoeur, I., Arensburg, B., Kaufman, D. & Ronen, A. Les hommes du Kébarien géométrique de Neve David, Mont Carmel (Israël). *Bull. Mem. Soc. Anthropol. Paris* **23**, 38–51 (2011).

78. Rolston, S. L. Two prehistoric burials from Qasr Kharaneh. *Annu. Dep. Antiq.* **26**, 221–229 (1982).

79. Stock, J. T., Pfeiffer, S. K., Chazan, M. & Janetski, J. F-81 skeleton from Wadi Mataha, Jordan, and its bearing on human variability in the Epipaleolithic of the Levant. *Am. J. Phys. Anthropol.* **128**, 453–465 (2005).

80. NESPOS Database. *NEanderthal Studies Professional Online Service* (2020). Available at: http://www.nespos.org.

81. Macchiarelli, R. *et al.* How Neanderthal molar teeth grew. *Nature* **444**, 748–751 (2006).

82. ESRF Paleonotological Microtomographic database. (2020). Available at: http://paleo.esrf.eu.

83. Bayle, P. *et al.* Dental maturational sequence and dental tissue proportions in the early Upper Paleolithic child from Abrigo do Lagar Velho, Portugal. *Proc. Natl. Acad. Sci. U. S. A.* **107**, 1338–1342 (2010).

84. Dibble, H. L. The Mousterian Industry from Bisitun Cave (Iran). *Paléorient* **10**, 23–34 (1984).

85. Trinkaus, E. & Biglari, F. Middle Paleolithic human remains from Bisitun Cave. *Paleorient* **32**, 105–111 (2006).

86. Bar-Yosef, O. *et al.* The Excavations in Kebara Cave, Mt. Carmel [and Comments and Replies]. *Curr. Anthropol.* **33**, 497–550 (1992).

87. Valladas, H. *et al.* Thermoluminescence dates for the Neanderthal burial site at Kebara in Israel. *Nature* **330**, 159–160 (1987).

88. Schwarcz, H. P. *et al.* ESR dating of the Neanderthal site, Kebara Cave, Israel. *J. Archaeol. Sci.* **16**, 653–659 (1989).

89. Volkman, P. & Marks, A. E. The Mousterian of Ksar Akil : levels XXVIA through XXVIIIB. **12**, 5–20 (1986).

90. Pagli, M. La equence de l’abri de Ksar ’Akil (Liban) et l’occupation du littoral mediterraneen du Proche-Orient pendant le Mousterien recent. in *Implantations humaines en milieu littoral méditerranéen: facteurs d’installation et processus d’appropriation de l’espace (Préhistorie, Antiqué, Moyen Age)* (eds. Mercuri, L., Villaescusa, R. G. & Bertoncello, F.) 177–190 (Association pour la promotion et la diffusion des connaissances archéologiques, 2014).

91. van der Plicht, J., van der Wijk, A. & Bartstra, G. J. Uranium and thorium in fossil bones: activity ratios and dating. *Appl. Geochemistry* **4**, 339–342 (1989).

92. Douka, K., Bergman, C. A., Hedges, R. E. M., Wesselingh, F. P. & Higham, T. F. G. Chronology of Ksar Akil (Lebanon) and Implications for the Colonization of Europe by Anatomically Modern Humans. *PLoS One* **8**, e72931 (2013).

93. Crew, H. L. The Mousterian Site of Rosh Ein Mor. in *Prehistory and Paleoenvironments in the Central Negev* (ed. Marks, A. E.) 75–112 (Southern Methodist University Press, 1977).

94. Goder-Goldberger, M. & Bar-Matthews, M. Novel chrono-cultural constraints for the Middle Paleolithic site of Rosh Ein Mor (D15), Israel. *J. Archaeol. Sci. Reports* **24**, 102–114 (2019).

95. Henry, D. (Ed. . *Neanderthals in the Levant*. (Continuum, 2003).

96. Henry, D. O. & Miller, G. H. The Implications of Amino Acid Racemization Dates of Levantine Mousterian Deposits in Southern Jordan. *Paléorient* **18**, 45–52 (1992).

97. Henry, D. O. The Prehistory of Southern Jordan and Relationships with the Levant. *J. F. Archaeol.* **9**, 417–444 (1982).

98. Yellen, J. *et al.* The Archaeology of Aduma Middle Stone Age Sites in the Awash Valley , Ethiopia. *PaleoAnthropology* **10**, 25–100 (2005).

99. Jennings, R. P. *et al.* Human occupation of the northern Arabian interior during early Marine Isotope Stage 3. *J. Quat. Sci.* **31**, 953–966 (2016).

100. Groucutt, H. S., Grün, R., Zalmout, I. S. A., Drake, N. A. & Simon, J. Homo sapiens in Arabia 85 , 000 years ago. 1–19 (2018).

101. Shea, J. J. The Middle Stone Age archaeology of the Lower Omo Valley Kibish Formation: Excavations, lithic assemblages, and inferred patterns of early Homo sapiens behavior. *J. Hum. Evol.* **55**, 448–485 (2008).

102. Groucutt, H. S. *et al.* Late Pleistocene lakeshore settlement in northern Arabia: Middle Palaeolithic technology from Jebel Katefeh, Jubbah. *Quat. Int.* **382**, 215–236 (2015).

103. Groucutt, H. S. *et al.* Human occupation of the Arabian Empty Quarter during MIS 5: Evidence from Mundafan Al-Buhayrah, Saudi Arabia. *Quat. Sci. Rev.* **119**, 116–135 (2015).

104. Hovers, E. *The Lithic Assamblages of Qafzeh Cave*. (Oxford University Press, 2009).

105. Groucutt, H. S., Scerri, E. M. L., Stringer, C. & Petraglia, M. D. Skhul lithic technology and the dispersal of Homo sapiens into Southwest Asia. *Quat. Int.* **515**, 30–52 (2019).

106. Usik, V. I., Ian, J., Hilbert, Y. H., Peer, P. Van & Marks, A. E. Nubian Complex reduction strategies in Dhofar , southern Oman. *Quat. Int.* **300**, 244–266 (2013).
